# Native earthworms and roe deer in temperate forests: Friends in the sun, foes in the shade

**DOI:** 10.1002/ecy.70520

**Published:** 2026-09-22

**Authors:** Ludwig Lettenmaier, Nico Eisenhauer, Simone Cesarz, Christian Ammer, Anna Bucharova, Sebastian Dittrich, Ronja Hausmann, Daniel Kraus, Angelika Kölbl, Soumen Mallick, Giorgi Mamadashvili, Atle Mysterud, Kerstin Pierick, Rike Schwarz, Jörg Müller, Christian Ristok

**Affiliations:** ^1^ Field Station Fabrikschleichach, Chair of Conservation Biology and Forest Ecology, Biocenter University of Würzburg Rauhenebrach Germany; ^2^ Bavarian Forest National Park Grafenau Germany; ^3^ German Centre for Integrative Biodiversity Research (iDiv) Halle‐Jena‐Leipzig Leipzig Germany; ^4^ Institute of Biology Leipzig University Leipzig Germany; ^5^ Silviculture and Forest Ecology of the Temperate Zones University of Göttingen Göttingen Germany; ^6^ Department of Biology Marburg University Marburg Germany; ^7^ Institute of General Ecology and Environmental Protection TUD Dresden University of Technology Tharandt Germany; ^8^ Universitätsforstamt University of Würzburg Haßfurt Germany; ^9^ Faculty of Environment and Natural Resources, Chair of Silviculture Albert‐Ludwigs‐University Freiburg Freiburg im Breisgau Germany; ^10^ Forest Protection Division Forest Maintenance and Reforestation Department, National Forestry Agency Tbilisi Georgia; ^11^ Centre for Ecological and Evolutionary Synthesis (CEES), Department of Biosciences University of Oslo Oslo Norway; ^12^ Spatial Structures and Digitalization of Forests, Faculty of Forest Sciences and Forest Ecology University of Göttingen Göttingen Germany

**Keywords:** above‐belowground interactions, browsing, canopy gap, disturbance, Lumbricidae, soil biodiversity, soil microbial biomass, soil pH, soil water content, ungulates, vegetation

## Abstract

Given increasing deer populations and rising canopy openness through tree mortality in European forests, understanding how these changes affect above‐belowground communities and ecosystem functioning is becoming more important. Earthworms comprise the largest animal biomass in European forest soils and play a central role in decomposition, carbon and nutrient cycling, and soil formation. However, their responses to canopy openness and deer browsing have been studied almost exclusively in northern North America, where interactions involve white‐tailed deer ( *Odocoileus virginianus* ) and mainly non‐native and invasive earthworms. Here, we examine how roe deer ( *Capreolus capreolus* ) and canopy openness interactively affect native earthworm communities in a temperate European forest across 75 experimental patches: 54 closed‐canopy and 21 experimentally created canopy‐gap patches. Five years after gap establishment, we sampled earthworms and associated environmental variables from two plots per patch: exclosure (fenced) and control (unfenced) plots. We found that roe deer and canopy gaps interactively influenced earthworm communities. Under closed canopy, roe deer reduced earthworm biomass and density. If roe deer were absent, canopy gaps had no effect on earthworms, but if roe deer were present, canopy gaps increased biomass and density through vegetation‐mediated pathways and enhanced soil water content. The magnitude of these effects differed among earthworm ecological groups (anecic species, i.e., earthworms that create deep, permanent burrows and connect surface litter with deeper soil layers, vs. endogeic species, i.e., earthworms that live and feed entirely within the soil) and life stages (juveniles vs. adults). Indirect effects of roe deer were particularly beneficial for juveniles. These results contrast with patterns reported for white‐tailed deer and non‐native earthworms in northern North American forests, where white‐tailed deer presence typically promotes earthworms. Our results on the direct and indirect effects of roe deer on belowground earthworms shed new light on the complex interactions of deer populations and canopy structure on forest soil biodiversity and ecosystem functioning.

## INTRODUCTION

Large herbivores are key drivers of forest ecosystem processes, shaping the flow of organic matter between above‐belowground compartments (Bardgett & Wardle, 2003). Through browsing and grazing, trampling, and deposition of feces and urine, they influence plant growth, plant community composition, and litter properties (Côté et al., 2004; Ramirez et al., 2020; Ribeiro et al., 2025). These effects cascade on belowground properties, leading to changes in nutrient mineralization, soil microbes, and decomposer activity (Bardgett & Wardle, 2003; Ribeiro et al., 2025). While many studies have examined the effects of large herbivores on soil processes (see Bardgett & Wardle, 2010; Ribeiro et al., 2025, and references therein), fewer have investigated their impacts on soil fauna, particularly earthworms.

Earthworms are key ecosystem engineers (Blouin et al., 2013; Jones et al., 1994) and comprise the largest animal biomass in European forest soils (Lavelle & Spain, 2001). Earthworms can be roughly classified into three ecological groups—anecic (deep‐burrowing surface feeders), epigeic (litter dwellers), and endogeic (soil dwellers) earthworms—based on their occupied niches as well as differences in feeding and burrowing strategies (Bouché, 1977; Edwards & Bohlen, 1996). In their native range, earthworms can show habitat preferences. For instance, of the 49 described earthworm species in Germany (Lehmitz et al., 2016), 10 species occur exclusively and 37 species occur transiently in forests (Graefe et al., 2019). Hence, earthworms are an essential part of soil biota communities in European forests. As such, they contribute to important ecosystem functions and services, such as decomposition, water regulation, soil aggregation, and nutrient cycling (Blouin et al., 2013; Wu et al., 2025). Shifts in their community composition and activity therefore can strongly affect forest structure, composition, functioning, and forest resilience (Lukac & Godbold, 2011).

Most studies on interactions between large herbivores and earthworms in forests have been carried out in northern North America and focused mainly on the browsing white‐tailed deer (*Odocoileus virginianus*). There, earthworm communities were largely eliminated by Pleistocene glaciation and have been re‐established only recently through introduction (Hendrix & Bohlen, 2002). Field studies comparing deer‐access and deer‐exclosure plots in northern North America generally report higher invasive earthworm biomass and density in plots accessible to white‐tailed deer than in deer exclosures (Cope & Burns, 2019; Dávalos et al., 2015; Mahon et al., 2020; Mahon & Crist, 2019; Reed et al., 2023), but also neutral effects (Dobson & Blossey, 2015; Shelton et al., 2014), and positive associations with native earthworms (Rearick et al., 2011). White‐tailed deer may promote earthworms due to fecal inputs and by reducing plant density, thereby increasing belowground resource availability, or by stimulating root allocation and thus soil microbial biomass following foliar loss (Bardgett & Wardle, 2003; Cope & Burns, 2019; Rearick et al., 2011; Reed et al., 2023). Other suggested mechanisms include disturbance of the litter layer and physical soil alterations through scrapes and trampling (Cope & Burns, 2019; Reed et al., 2023). Such browsing‐induced changes may also affect soil pH and soil water content (Harrison & Bardgett, 2004; Ribeiro et al., 2025), with soil pH shown to correlate with browser–earthworm interactions (Dávalos et al., 2015), and soil moisture representing a key abiotic control of earthworm performance (de Wandeler et al., 2016; Singh et al., 2019). Outside of North America, studies linking browsers and earthworms are scarce. Although earthworms have been included in broader soil faunal assessments in Europe (e.g., Ramirez et al., 2020), explicit tests of browser effects on earthworms are rare, with the only evidence from the mid‐20th century suggesting negative effects of roe deer (*Capreolus capreolus*) on native earthworm density (Sommer, 1956).

The impact of deer and forest disturbances in combination on earthworm populations is even less well understood. Forest disturbances, such as silvicultural interventions, windthrows, pest outbreaks, or droughts, frequently lead to partial or complete tree mortality and thereby to the formation of canopy gaps (Seidl et al., 2017). These gaps alter forest microclimate (Pierick, Seidel, et al., 2026; Ritter et al., 2005), reduce soil carbon while increasing nutrient availability (Tong et al., 2024), and promote understory vegetation richness (Kern et al., 2014) as well as woody plant regeneration (Zhu et al., 2014). Changes in leaf litter properties, soil chemistry, and plant composition strongly influence earthworm presence in forests (Ammer et al., 2006; de Wandeler et al., 2016). The direct effect of canopy openness on earthworms varies between studies from positive to negative (Ganault et al., 2021; Nachtergale et al., 2002; Reed et al., 2023). To our knowledge, only one northern North American study has combined deer exclosures with canopy gap manipulation. Building on evidence that deer may preferentially use canopy gaps (Kuijper et al., 2009), Reed et al. (2023) hypothesized that canopy gaps would increase white‐tailed deer activity and thereby indirectly promote earthworm populations. Their results attributed positive effects of white‐tailed deer on invasive earthworms primarily to indirect mechanisms via vegetation changes, leading to increased soil microbial biomass and soil nutrient availability, whereas canopy gaps reduced earthworm populations by creating less favorable leaf litter conditions.

In the context of global change, understanding the interaction between herbivores, canopy gaps, and earthworms in temperate forests is particularly important. Deer populations are rising worldwide, including white‐tailed deer in North America (Côté et al., 2004), and roe deer in Europe, the locally dominant browser in most European forests (Burbaitė & Csányi, 2009; Ionescu et al., 2025). Simultaneously, canopy dieback is increasing in many European temperate forests (Senf et al., 2018), and future light regimes are expected to shift under global change, affecting biodiversity and ecosystem functions (de Frenne, 2024). These trends highlight the need to understand how deer and canopy gaps affect earthworms, given earthworms' pivotal role in sustaining forest resilience (Byers et al., 2006). However, distinct biogeographic and evolutionary contexts raise uncertainty about the extent to which results from northern North America are transferable to European forests, where earthworms are native and have long co‐evolved with plants, soils, and large herbivores. In addition, white‐tailed deer are nearly three times larger than roe deer despite having broadly similar browsing strategies (Loison et al., 1999; van Wieren, 1996).

**FIGURE 1:**
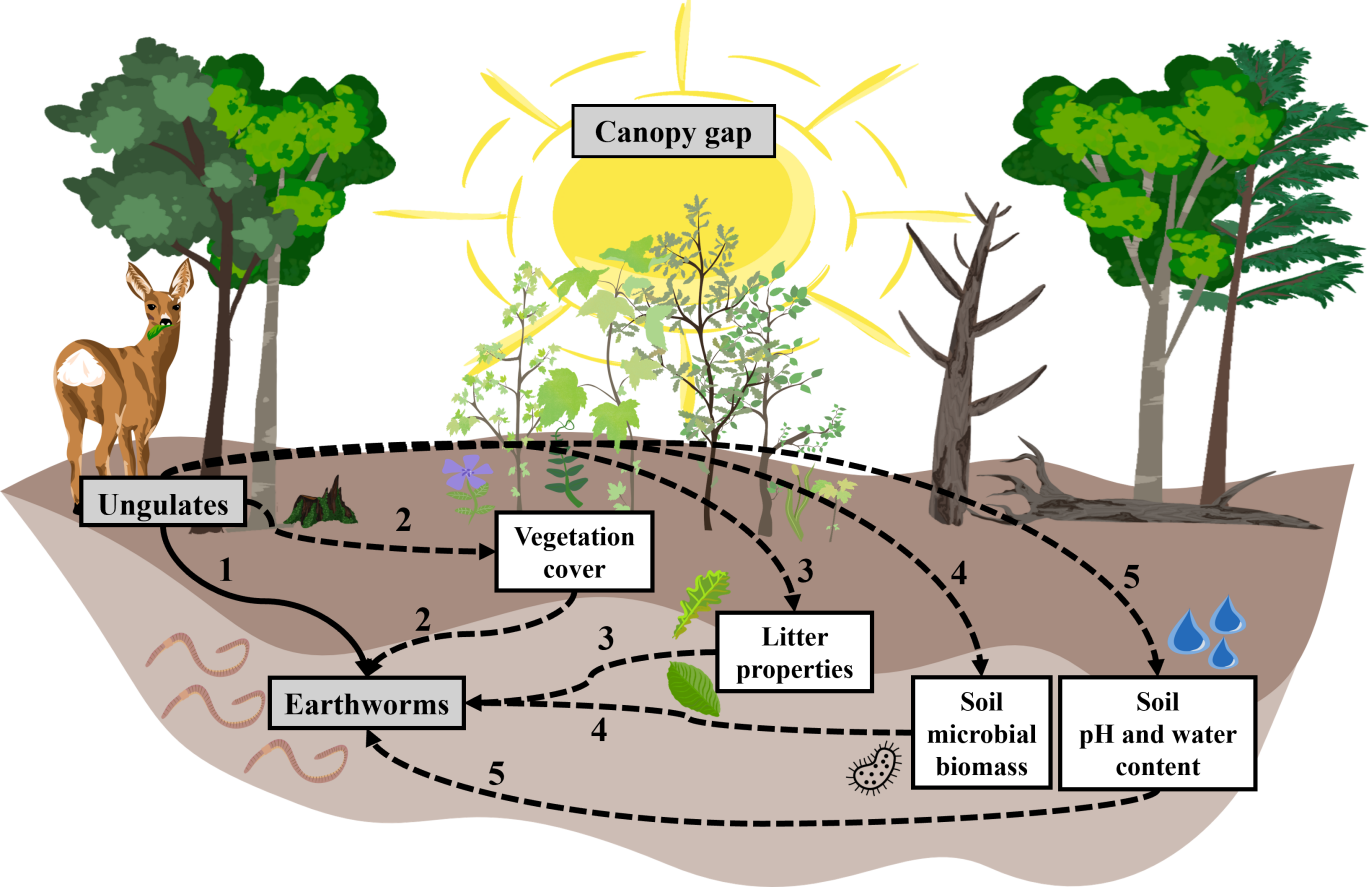
Simplified conceptual illustration showing commonly described mechanisms (1–5; see *Introduction*) by which ungulates may influence earthworm populations, depending on canopy condition (closed canopy and canopy gap). The solid arrow indicates the direct pathway, whereas dashed arrows indicate indirect pathways. Mechanisms include (1) direct effects via fecal input and soil disturbance (trampling), and indirect effects via (2) vegetation structure, (3) litter properties, (4) soil microbial biomass, and (5) soil pH and soil water content. Figure created by Rabea Klümpers.

To address the open questions of the deer–canopy–earthworm relationship, we conducted a replicated, full‐factorial field experiment comparing paired control (roe deer presence) and exclosure (roe deer absence) plots in closed‐canopy forests and experimentally created canopy gaps in Germany. Because roe deer and canopy gaps can alter vegetation structure, litter conditions, microbial biomass, soil pH, and soil water content, we expected their effects on earthworms to be context‐dependent (Figure 1). Based on Reed et al. (2023), we hypothesized: (H1) roe deer increase and (H2) canopy gaps decrease earthworm biomass and density. (H3) Roe deer mitigate the negative effects of canopy gaps on earthworm populations. (H4) Roe deer affect earthworm populations through direct pathways beyond the measured environmental variables and through indirect pathways mediated by vegetation cover, litter depth, soil microbial biomass, soil pH, and soil water content, with these pathways differing between closed‐canopy forests and canopy gaps.

## METHODS

### Study location and experimental design

The experiment was carried out in the University Forest of the Julius‐Maximilians‐University Würzburg, Germany (50°04′ N, 10°26′ E). This managed mixed forest covers 2176 ha and consists mainly of European ash (*Fraxinus excelsior*), European beech (*Fagus sylvatica*), maple (*Acer* sp.), hornbeam (*Carpinus betulus*), and oak (*Quercus* sp.), with smaller proportions of other broadleaved species and occasional conifers such as pine (*Pinus* sp.), Douglas fir (*Pseudotsuga menziesii*), and European larch (*Larix decidua*). The soils are calcareous loams (Eutric Chromic Endoleptic Cambisol according to the World Reference Base for Soil Resources, Leitgeb et al., 2013). Roe deer is the only large herbivore present in the forest, occurring at densities of 20–30 individuals km^−2^ (Lettenmaier et al., 2025). During the winter of 2018–2019, two types of canopy manipulation were implemented to enhance structural diversity and heterogeneity by removing approximately 30% of trees with a diameter at breast height (dbh) >7 cm from each manipulated forest patch (patch size: 50 × 50 m) (see Müller et al., 2023 for details). Aggregated tree removal created 21 forest patches with canopy gaps (~30 m in diameter), whereas distributed tree removal was applied across 51 patches and resulted in only a slight opening of the closed canopy. Three patches remained untreated and served as controls for the structural diversity and heterogeneity experiment (Müller et al., 2023). Due to the rapid canopy closure within one year, patches subjected to distributed tree removal became structurally indistinguishable from untreated patches (Pierick, Link, et al., 2026). These patches were therefore pooled, resulting in 54 closed‐canopy patches (patch size: 50 × 50 m). In May 2019, a paired plot design was established on each of the 75 patches. Each patch contained one fenced plot excluding roe deer (wildlife fence; 6 m × 6 m area, 2 m in height) and one unfenced control plot, allowing assessment of roe deer effects under both canopy conditions. One of these two plots was positioned in the center of the patch, while the other was located 10 m away. The assignment of whether the central position was fenced or unfenced was made randomly by tossing a coin. This resulted in a spatial explicit paired design with four treatment combinations: (1) canopy gap and roe deer presence (*n* = 21), (2) canopy gap and roe deer absence (*n* = 21), (3) closed‐canopy and roe deer presence (*n* = 54), and (4) closed‐canopy and roe deer absence (*n* = 54). Roe deer browsing pressure was not quantified, as we assumed that roe deer had unrestricted access to all unfenced control plots.

### Earthworms

Earthworm sampling was conducted over 9 consecutive days between 24 September and 3 October 2024. In each plot, a soil block measuring 25 cm × 25 cm area, 10 cm in depth was excavated from an area representative of the dominant vegetation. The soil was hand‐sorted for earthworms, which were then collected and preserved in 70% ethanol. To extract earthworms inhabiting deeper soil layers, 5 L of mustard solution (10 g mustard powder L^−1^ water) was poured into each excavated hole. Emerging earthworms were collected for 15 min, rinsed with water, and transferred to 70% ethanol. This procedure was repeated with a second 5‐L application, followed by a further 15‐min collection period (Ganault et al., 2024). Individuals were classified as either juveniles or adults according to the presence or absence of a clitellum and tubercula pubertatis and assigned to ecological groups (anecic, epigeic, or endogeic). Earthworms were counted, biomass weighed, and if possible, identified to species level using morphological characters, including segment counts (Brohmer & Schaefer, 2010; Sherlock, 2018). Identifications were cross‐referenced with information on forest affinity of earthworm species in Germany (Graefe et al., 2019). If species‐level identification was not possible, ecological group assignment was based on morphological characteristics. Large, juvenile, partially pigmented earthworms were assigned to *Lumbricus terrestris*, the only anecic species occurring in the study area. All other darkly pigmented earthworms were classified as epigeic, whereas grayish to pale‐pink earthworms were classified as endogeic (Bouché, 1977; Brohmer & Schaefer, 2010; Sherlock, 2018).

### Environmental data

Vegetation was surveyed in June 2024 and recorded separately for two strata: (i) the shrub layer, defined as woody species (1–6 m in height), and (ii) the herb layer, defined as vascular plants <1 m in height, excluding bryophytes and lichens. For each stratum, cover was estimated as a percentage of 2 m × 2 m in the treatment plot center using a simplified cover‐abundance approach following Dittrich et al. (2013). In 2019, all trees with a dbh >7 cm within each forest patch (50 m × 50 m) were inventoried. Tree species, stem coordinates, and dbh were recorded. Litter depth was measured with a ruler at four points (from the center 50 cm in each cardinal direction) as the distance between the upper litter surface and the mineral soil. The mean per plot was used for further analyses. Litter quality was estimated using litter carbon‐to‐nitrogen (C:N) ratios of the overstory tree species present in each patch, derived from the TRY plant trait database (Kattge et al., 2020) for all overstory tree species recorded in the patches (50 m × 50 m). Details of the calculation are provided in Appendix S1. For soil pH determination, soil samples collected during earthworm extraction were dried in an oven at 30°C for 5 days and subsequently sieved through a 4‐mm mesh. Soil pH was measured in a 1:2.5 CaCl_2_ solution (0.01 mol L^−1^) using 10 g of the sieved soil (<4 mm fraction) with an Orion Star A211 pH benchtop meter equipped with an Orion ROSS Ultra Triode pH/ATC combination electrode (Thermo Scientific). To quantify soil microbial biomass and soil water content, three soil cores per plot were collected using a steel corer (Ø = 2 cm, depth = 10 cm) to account for spatial heterogeneity. All samples per plot were pooled, sieved through a 2‐mm sieve, and stored at 4°C until soil microbial biomass measurement using an O_2_‐microcompensation system (Scheu, 1992). Approximately 7 g of sieved soil per plot were transferred into glass pots and, following a 24‐h incubation period at 20°C, glucose (8 mg g^−1^ soil dry mass, dissolved in 1.25 mL distilled water) was added to each pot. Subsequently, we assessed the maximum initial respiratory response (MIRR), a proxy for active soil microbial biomass (in micrograms of microbial carbon per gram of soil; soil dry mass, equal to 38 × MIRR; Beck et al., 1997), as the lowest substrate‐induced respiration observed over three consecutive hours within the first 10 h. Soil water content was determined gravimetrically by drying samples at 105°C for 24 h and calculating the difference between fresh and dry soil mass, expressed as a percentage of fresh mass.

### Statistical analysis

Statistical analysis was performed in R version 4.3.1 (www.r-project.org). For analysis of earthworm density, only intact individuals were included to avoid multiple counts. Biomass analyses included both uncut and cut individuals. We tested our treatments on earthworm biomass and density using generalized linear mixed‐effects models (LMMs) (Hypotheses 1–3). In all models, canopy condition (closed canopy vs. gap) and roe deer (absence vs. presence), and their interaction were included as fixed effects, with closed‐canopy and roe deer absence plots set as reference levels. Patch was included as a random effect to account for the paired design. Soil pH and soil water content were added as scaled covariates to control for soil variability. Earthworm biomass was cube‐root transformed to improve model fit and analyzed using Gaussian distribution (LMM). The count data density was analyzed with Gaussian linear mixed‐effects models (GLMMs) using a negative binomial distribution with a log link. To test whether the effect of roe deer presence and canopy gap on biomass and density depends on the life stage (juveniles and adults) and ecological group (including only anecic and endogeic, as they were represented by enough individuals, see Appendix S2: Table S1), we fitted models including the main effect of the focal factor (life stage or ecological group) and its interactions with canopy, roe deer, their combined interaction, and scaled soil pH and soil water content. Biomass was modeled using LMMs (cube‐root transformed) for life stages and Tweedie GLMMs (log link) for ecological groups, and densities were analyzed using negative binomial GLMMs. Models were implemented using either the *lme4* (Bates et al., 2015) or *glmmTMB* (Brooks et al., 2017) package. Optimizer settings were adjusted where necessary (e.g., bobyqa) to ensure convergence. Model residuals were checked using DHARMa (Hartig, 2022), but no substantial problems with overdispersion, zero‐inflation, or residual structure for any (G)LMMs could be found. To test the overall significance of our canopy and roe deer treatments, as well as their interaction, and the environmental covariates, we applied Type III Wald χ^2^ tests using the Anova function in the *car* package (Fox & Weisberg, 2018). When significant or marginal significant interactions involving canopy and roe deer were detected, either in the overall earthworm models or in models including life stage or ecological group, we evaluated planned contrasts among the treatment levels and their combinations using general linear hypothesis tests (glht) with Holm‐adjusted *p*‐values implemented in the *multcomp* package (Hothorn et al., 2008). Additional analyses examining how consumable resources, such as soil microbial biomass and litter depth, responded to experimental treatments and earthworms 5 years after treatment establishment are presented in Appendix S3.

Structural equation models were subsequently used to explore potential direct and indirect mechanisms that may underlie the overall effects of deer presence and canopy gaps (Hypothesis 4; Figure 1; Appendix S4: Figure S1, Table S1). Specifically, we applied a piecewise structural equation modeling (pSEM) framework based on LMMs that link canopy gaps, roe deer presence, and their interaction with earthworms. The pSEM quantified how roe deer presence under different canopy conditions directly affected earthworms (via fecal input and trampling), Mechanism (1); indirectly via changes in vegetation cover, Mechanism (2); litter depth, Mechanism (3); soil microbial biomass, Mechanism (4); soil pH and soil water content, Mechanism (5), and how these variables in turn influenced earthworm biomass, density, life stages, and ecological groups. Litter quality was included only as a background covariate, because roe deer were unlikely to have caused measurable changes in overstory tree species composition within 5 years of treatment establishment. All component models included canopy gap, roe deer presence, and their interaction as predictors, and patch was included as a random effect to account for the non‐independence of the paired fenced and non‐fenced plots within one patch. Residual covariance between soil pH and soil water content was included to account for shared edaphic influences. All response variables were standardized prior to analysis; proportional variables were arcsine‐square‐root transformed, and litter quality (C:N ratio) was inverted so that lower values indicate higher litter quality. Individual mixed models were combined into pSEMs using the *piecewiseSEM* package (Lefcheck, 2016). Because the pSEMs were close to saturated, global tests of directed separation were limited. Inference therefore focused on standardized path coefficients of the component models (Appendix S4: Table S2) and on the decomposition of direct and indirect effects (Appendix S4: Table S3). Standardized path coefficients were decomposed into direct, indirect, and net effects of roe deer presence on earthworm responses. Indirect effects were calculated as the sum of products of standardized coefficients along all directed pathways linking roe deer presence to earthworms via vegetation (shrub and herb layers), litter depth, and soil (microbial biomass, pH, and water content). Canopy‐dependent effects were derived using a simple‐slope approximation based on the canopy × roe deer interaction terms, yielding separate estimates for closed canopy forest patches and for canopy gaps (Appendix S4). See Appendix S4: Figure S1, Table S1 for the full set of modeled paths and the literature supporting each hypothesized relationship.

## RESULTS

In total, we collected 1509 earthworm individuals, of which 1196 were juvenile and 313 were adults. Endogeic earthworms were most abundant (947 individuals), followed by anecic (550 individuals) and epigeic (8 individuals) earthworms (Appendix S2: Table S1). Environmental variables (soil pH, soil water content, litter depth and quality, soil microbial biomass, and shrub and herb layers cover) were weakly to moderately correlated, with absolute Spearman rank correlations ranging from 0.03 to 0.51 (Appendix S5: Figure S1). Total earthworm biomass and density were strongly correlated (Spearman's ρ = 0.61, *p* < 0.001).

### Treatment effects on earthworms

**FIGURE 2:**
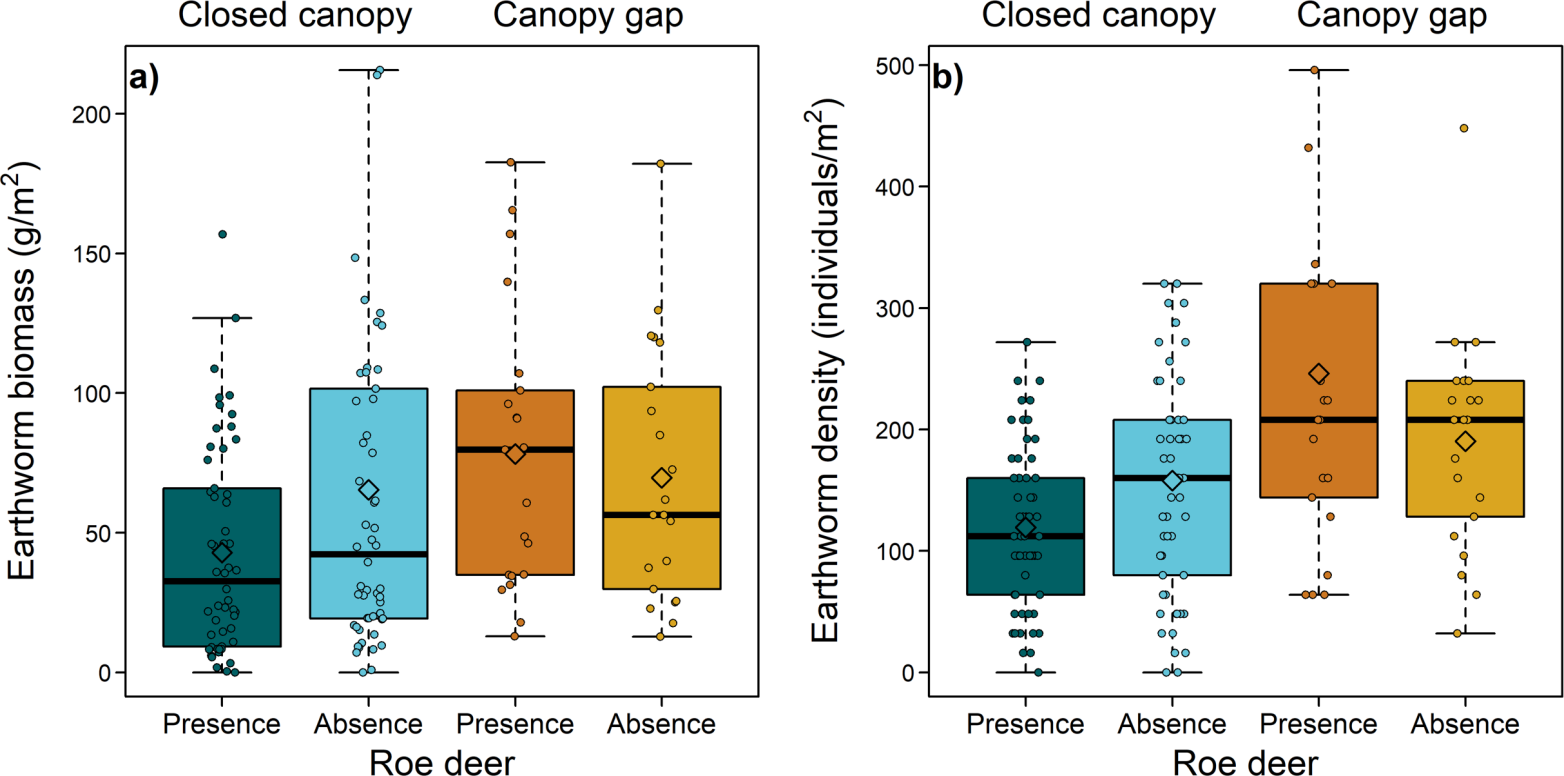
Total earthworm (a) biomass (in grams per square meter) and (b) density (in individuals per square meter) across canopy conditions (closed canopy vs. canopy gap) and roe deer (presence vs. absence) treatments. Roe deer presence and absence plots were established in canopy gaps (*n* = 21 per treatment) and closed canopy forest patches (*n* = 54 per treatment). Boxplots show medians, interquartile ranges, and whiskers. Circles show raw data, and diamonds indicate group means. Statistical tests for planned contrasts among treatment combinations are reported in Table 2.

Earthworm biomass and density were both significantly affected by roe deer treatment, but there was no significant main effect of canopy condition. However, the canopy × roe deer interaction was marginally significant for biomass and significant for density, indicating that the effect of roe deer presence depended on canopy condition (Table 1). Planned contrasts revealed that under closed‐canopy conditions, roe deer presence significantly reduced total earthworm biomass (−33%) and marginally reduced total density (−22%). In contrast, roe deer presence had no detectable effect on earthworms in canopy gaps. Conversely, canopy gaps increased earthworm biomass (+15%) and density (+21%) only when roe deer were present, whereas canopy condition had no detectable effect when roe deer were absent (Figure 2, Table 2). Higher soil pH increased total biomass and showed a marginal positive effect on density, whereas soil water content was not significant (Appendix S6: Table S1).

**TABLE 1:** Type III Wald χ^2^ tests evaluating the effects of canopy condition, roe deer, and their interaction and soil covariates on total earthworm biomass and density.

	df	χ^2^	*p*‐value	_marg_ *R* ^2^	_cond_ *R* ^2^
Earthworm biomass				0.12	0.38
Canopy	1	0.33	0.566		
Roe deer	1	5.77	0.016		
Canopy × Roe deer	1	3.00	0.083		
Soil pH	1	3.94	0.047		
Soil water content	1	0.20	0.657		
Earthworm density				0.20	0.40
Canopy	1	0.63	0.427		
Roe deer	1	5.71	0.017		
Canopy × Roe deer	1	5.25	0.022		
Soil pH	1	3.68	0.055		
Soil water content	1	2.39	0.122		

*Note*: Biomass was analyzed using linear mixed‐effects models. Density was analyzed using negative binomial generalized linear mixed‐effects models. Models included scaled soil pH and soil water content as covariates and patch as a random effect. χ^2^ values, df, *p*‐values, and marginal and conditional *R* ^2^ are shown.

**TABLE 2:** Planned contrasts among four treatment combinations testing context‐dependent effects of roe deer and canopy condition on total earthworm biomass and density.

Contrast	Estimate	SE	95% CI lower	95% CI upper	*z*‐value	*p*‐value
Earthworm biomass						
Closed canopy: Roe deer presence–absence	−0.182	0.076	−0.330	**−**0.033	−2.402	0.049
Canopy gap: Roe deer presence–absence	0.073	0.123	−0.168	0.314	0.594	1.000
Roe deer absence: Canopy gap–closed canopy	0.068	0.119	−0.164	0.300	0.574	1.000
Roe deer presence: Canopy gap–closed canopy	0.323	0.125	0.078	0.567	2.587	0.039
Earthworm density						
Closed canopy: Roe deer presence–absence	−0.253	0.106	−0.460	−0.045	−2.389	0.051
Canopy gap: Roe deer presence–absence	0.194	0.160	−0.119	0.506	1.214	0.450
Roe deer absence: Canopy gap–closed canopy	0.123	0.155	−0.180	0.426	0.795	0.450
Roe deer presence: Canopy gap–closed canopy	0.569	0.163	0.250	0.889	3.491	0.002

*Note*: Planned contrasts were derived from (generalized) linear effect models. Estimates represent differences between treatment levels within canopy or roe deer context. SEs, 95% CIs, *z*‐values, and Holm‐adjusted *p*‐values are reported.

The effect of roe deer on earthworm biomass and density differed between life stages, as indicated by significant roe deer presence × life stage interaction. Additionally, the marginally significant three‐way interaction roe deer × life stage × canopy condition for density suggests that the roe deer effect on density of life stages is further modulated by canopy gaps (Appendix S6: Table S2). Planned contrasts showed that roe deer presence significantly reduced adult earthworm biomass and density under closed‐canopy conditions, but no roe deer effect was detected in canopy gaps. For juveniles, canopy gaps significantly increased density, but not biomass, only when roe deer were present (Appendix S6: Table S3, Figure S1).

The effect of roe deer further differed between ecological groups, as indicated by significant roe deer × ecological group interaction. The three‐way interaction with canopy was marginal for biomass but significant for density (Appendix S6: Table S2). Planned contrasts revealed that roe deer presence significantly reduced anecic biomass and density under closed‐canopy conditions, whereas no effect was detected for endogeic earthworms. Under roe deer presence, canopy gaps marginally increased anecic biomass and significantly increased the density of both anecic and endogeic earthworms, but no effect of canopy gaps was found when roe deer were absent (Appendix S6: Table S3, Figure S2).

### Direct and indirect effects of roe deer on earthworms across canopy conditions

**FIGURE 3:**
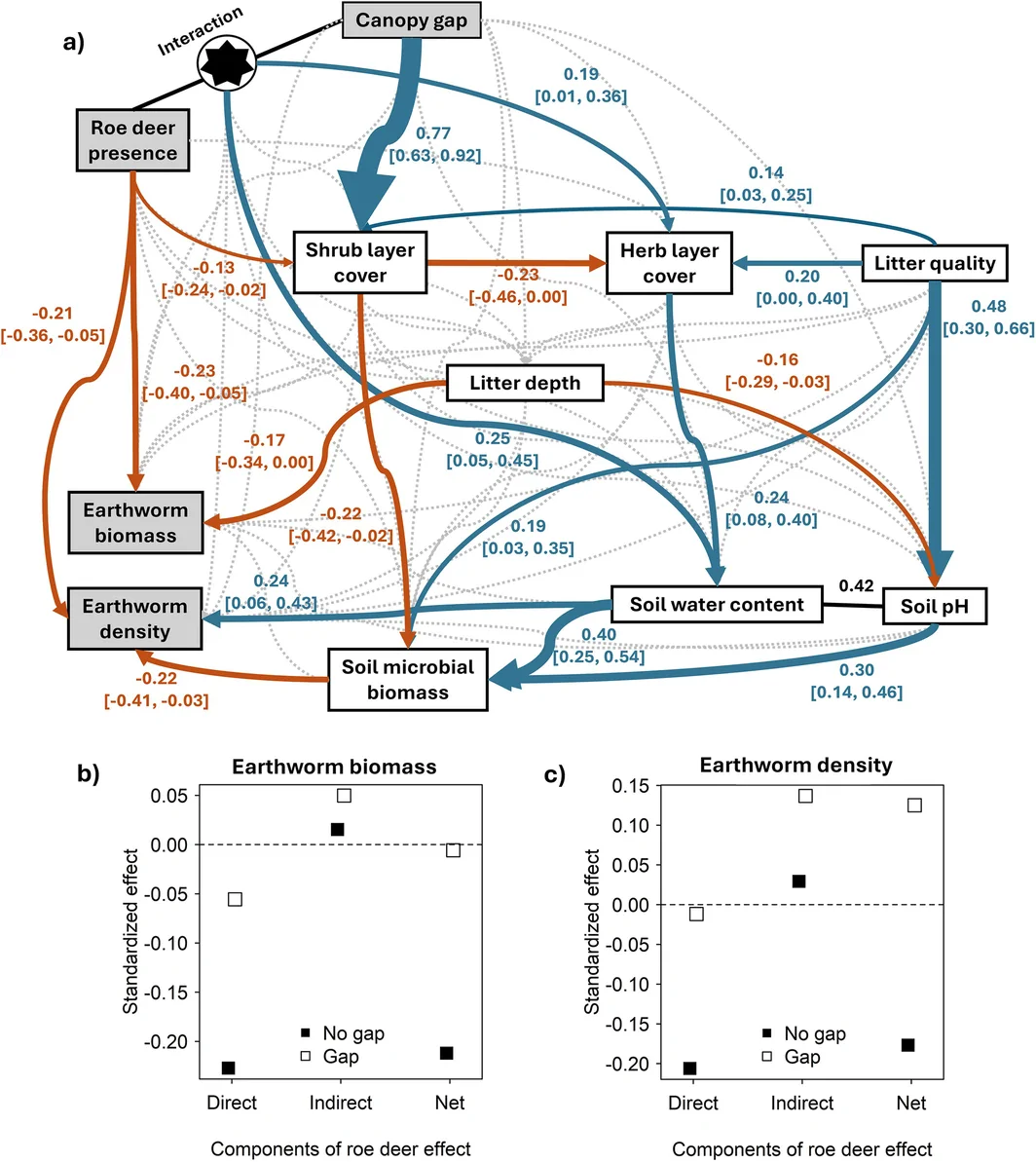
(a) Piecewise structural equation model (pSEM) illustrating pathways linking canopy gaps, roe deer presence, shrub and herb layer cover, litter depth and quality (C:N ratio), soil pH, soil water content, and soil microbial biomass to total earthworm biomass and density. Arrow labels show standardized path coefficients with approximate 95% CIs in brackets. Paths are color‐coded by effect direction (blue = significant positive, orange = significant negative, gray dashed = nonsignificant). The black line indicates covariance among soil pH and soil water content, and the black line with a star denotes the canopy × roe deer interaction. Arrow thickness is proportional to standardized path coefficients. Standardized direct, indirect, and net effects of roe deer presence on total earthworm (b) biomass, and (c) density are derived from the pSEMs. Filled symbols represent closed canopy patches, and open symbols represent patches with canopy gaps. Net effects equal the sum of standardized direct and indirect effects. Direct, indirect, and net effects are based on all modeled paths, including nonsignificant ones. Full unstandardized and standardized pSEM coefficients, including 95% CIs, test statistics, df, and *p*‐values, are reported in Appendix S4: Table S2.

We report the decomposed direct, indirect, and net effects of roe deer presence on earthworm biomass and density across canopy conditions (Figure 3b,c; Appendix S4: Table S3). Detailed results of the underlying structural equation models are presented in Appendix S4: Table S2 and illustrated in Figure 3a, and in Appendix S4: Figures S2–S5. In closed‐canopy forests, roe deer presence exerted strong negative direct effects on total earthworm biomass (−0.227) and total density (−0.206). These direct effects were partly counterbalanced by positive indirect effects (biomass: 0.015; density: 0.029), resulting in net negative effects on total earthworm biomass (−0.212) and density (−0.177) (Figure 3b,c; Appendix S4: Table S3). In canopy gaps, direct effects of roe deer presence on total earthworm biomass (−0.056) and total density (−0.012) were weaker. Indirect effects were stronger than in closed‐canopy forests (biomass: 0.050; density: 0.137), resulting in near‐neutral net effects on total biomass (−0.006) and positive net effects on total density (0.125) (Figure 3b,c; Appendix S4: Table S3). Generally, positive indirect effects arose because roe deer reduced shrub layer cover, which in turn increased herb layer cover. Increased herb layer cover promoted soil water content, which in turn had positive effects on earthworms. In addition, soil microbial biomass, decreased by shrub layer cover and increased by litter quality, soil pH, and soil water content, was negatively related to total earthworm density (Figure 3a; Appendix S4: Table S2).

Decomposition of roe deer effects revealed consistent canopy‐dependent patterns across earthworm life stages and ecological groups. In closed‐canopy forests, roe deer presence consistently exerted negative direct effects on biomass and density of juveniles, adults, anecic, and endogeic earthworms (Appendix S4: Table S3). Indirect effects were generally positive but small. As a result, net effects on biomass and density were negative across all groups in closed‐canopy forests. In contrast, canopy gaps showed positive net effects on juvenile biomass and density, as well as on anecic and endogeic density (Appendix S4: Table S3). Juvenile earthworms benefited mainly from increased herb layer cover, soil water content, and soil pH, whereas adult earthworms were more constrained by roe deer presence and litter depth (Appendix S4: Table S2, Figures S2 and S3). Anecic earthworms showed pronounced negative responses to roe deer presence and litter depth, while endogeic earthworms responded positively to canopy gaps, herb layer cover, and soil water content, but negatively to soil microbial biomass (Appendix S4: Table S2, Figures S4 and S5).

## DISCUSSION

Both large herbivores and earthworms fulfill important functional roles in ecosystems, yet their interlinkages in European deciduous forests remain poorly understood. Contrary to our expectations derived from studies in northern North America, roe deer presence in European temperate forests did not generally increase earthworm biomass or density (H1), nor did canopy gaps reduce earthworm populations (H2). Instead, roe deer presence and canopy gaps interacted strongly, partially supporting H3. Roe deer reduced earthworm populations in closed‐canopy forests, whereas canopy gaps increased earthworm populations if roe deer were present. This interaction reflects complex, context‐dependent effects of roe deer presence and canopy gaps on environmental conditions, highlighting how roe deer can both suppress and enhance, at least temporarily, earthworm populations depending on the forest structure (supporting H4).

### Roe deer decrease earthworm populations in closed‐canopy forests

Dense, closed‐canopy forests with roe deer as the dominant ungulate species represent the current status quo in many European forests (Burbaitė & Csányi, 2009; Ionescu et al., 2025; Müller et al., 2023). Under these dominant conditions, roe deer presence reduced total earthworm biomass and density, which is consistent with early anecdotal observations from a Central European forest (Sommer, 1956). However, this study, conducted nearly 75 years ago, did not account for variation in canopy density, and the lack of replication limits the reliability of its conclusions. Our results contrast with studies from northern North America reporting predominantly positive effects of white‐tailed deer on invasive earthworms (Cope & Burns, 2019; Dávalos et al., 2015; Fisichelli & Miller, 2018; Mahon et al., 2020; Mahon & Crist, 2019; Reed et al., 2023). Comparisons between North American and European studies must be made with caution, as forest ecosystems differ substantially (e.g., differently thick litter layers), and because most northern North American studies investigated non‐native or invasive earthworms rather than native, co‐adapted deer‐earthworm systems.

The deer effects on earthworms have often been attributed to direct soil‐related processes (Mechanism 1, Figure 1), including fecal nutrient inputs and trampling‐induced soil disturbance. While dung deposition can locally enhance soil fauna including earthworms (Bacher et al., 2018; Holter, 1983; Singh et al., 2021) and cervid feces can promote certain earthworm species (McAlpine et al., 2025; Rearick et al., 2011), trampling may compact soil (Ramirez et al., 2020) and reduce habitat suitability, particularly for larger‐bodied earthworms (Jordan et al., 2000). In our study, structural equation models showed strong negative direct effects of roe deer, particularly under closed‐canopy, which suggests that physically mediated processes dominate here. Additionally, direct negative effects were stronger for adult than for juvenile earthworms, indicating life‐stage‐specific sensitivity. Under closed‐canopy conditions, soils typically exhibit well‐developed and persistent structure, and trampling by large herbivores can disrupt established macropores and increase soil compaction, with particularly strong consequences for large‐bodied adult earthworms that rely on persistent macropores (Piearce, 1984). Juvenile earthworms, which are smaller, more mobile, and exploit shallow soil layers, appear more resilient to such physical disturbance and respond more strongly to indirect, gap‐mediated increases in herb layer cover and soil water content (Gasch et al., 2021; Piz̆l, 1992) (see below). Together, these patterns indicate that in closed‐canopy forests, roe deer likely impose physical constraints on earthworm populations that disproportionately affect adults.

### Canopy gaps transform roe deer–earthworm interactions

Canopy gaps increased earthworm biomass and density, especially juvenile density, but only when roe deer were present. This contrasts with the results of Nachtergale et al. (2002), who observed a decline in earthworm biomass in disturbed gaps compared with closed‐canopy soils. The discrepancy suggests that canopy opening alone may not substantially reduce earthworm populations unless accompanied by direct soil disturbance. Reed et al. (2023) also reported declines in invasive earthworm biomass and density in canopy gaps, emphasizing differences in how native and invasive species in different forest ecosystems can respond to disturbances. Importantly, the increase in earthworm biomass and density in canopy gaps in the presence of roe deer is likely driven by interactive changes in environmental conditions.

Canopy gaps indirectly reduced earthworm density by promoting the shrub layer, which suppressed the herb layer, resulting in a lower soil water content, which was positively associated with earthworms. In contrast, roe deer presence was associated with reduced shrub layer cover, most likely due to browsing, partially counteracting these gap‐related pathways. Furthermore, our results suggest that endogeic and juvenile earthworms were positively associated with herb layer development. Similar interactions between deer browsing and earthworms have been reported for invasive earthworms with white‐tailed deer, suggesting that endogeic species are closely linked to the root zone of the herb layer and therefore respond more directly and rapidly to browsing and natural regeneration than to overstory tree composition (Cope & Burns, 2019). For juvenile earthworms, we postulate that this positive association may reflect more favorable near‐ground conditions in plots with higher herb layer cover, for example through greater fine‐root and labile organic inputs. However, because we did not directly measure hatchling survival or fine‐root inputs, this mechanism remains a plausible interpretation rather than a directly tested pathway. Although we did not analyze species‐specific vegetation responses in the present study, canopy‐gap vegetation was not dominated by a single plant species. The shrub layer mainly consisted of regenerating broadleaved tree species, particularly *Acer campestre*, *A. pseudoplatanus*, *C. betulus*, and *F. excelsior*, whereas high local herb‐layer cover was distributed among several species, including *Dactylis glomerata*, *Melica uniflora*, and *Urtica dioica*. Previous work at the same experimental site showed that roe deer reduced the diversity of woody regeneration in canopy gaps by half (Lettenmaier et al., 2025), likely contributing to the lower shrub‐layer cover observed under roe deer presence. Together, these findings provide support for Mechanism 2 (Figure 1), whereby changes in vegetation structure mediate herbivore effects on earthworms (Bardgett & Wardle, 2003).

Litter depth was negatively associated with total earthworm biomass. Specifically, adult biomass and anecic density declined with increasing litter depth. At the same time, litter depth was lower in canopy gaps than in closed forest patches, and it was only weakly reduced by roe deer presence (Appendix S3: Table S1). This pattern contrasts with the results from long‐term studies from Europe and North America, which reported reduced litter accumulation under increased ungulate browsing (Bressette et al., 2012; Ramirez et al., 2020). In our experiment, canopy gaps (~30 m in diameter) within the experimental forest patches (50 m × 50 m) represent early‐stage regenerating stands, with trees not exceeding 7 m in height 5 years after gap creation. Here, litter depth is likely primarily driven by the overstory trees that are unaffected by roe deer presence. Early changes in litter accumulation following roe deer presence during initial tree regeneration likely play only a minor role in determining litter depth, and thus in influencing earthworms. Consequently, this finding does not support Mechanism 3 (Figure 1) that roe deer directly or indirectly alter litter depth in early successional stages of temperate forests, but instead suggests that canopy gap is the dominant driver of litter dynamics (Ganault et al., 2021; Nachtergale et al., 2002; Zhang et al., 2024).

Soil microbial biomass was negatively associated with total earthworm density, with endogeic earthworm biomass and density declining as soil microbial biomass increased. In addition, soil microbial biomass was lower in canopy gaps than in closed‐canopy forest patches, independent of roe deer presence (Appendix S3: Table S1). The effects of canopy disturbance (i.e., canopy gap formation) on soil microbial communities are highly variable, with positive, negative, or negligible responses depending on disturbance intensity, forest type, and biome (Pérez‐Izquierdo et al., 2025; Tong et al., 2024; Wu et al., 2019). In our study, roe deer presence limited shrub layer development, and thus, was indirectly associated with higher soil microbial biomass. This pattern is consistent with Mechanism 4 (Figure 1), which proposes that herbivory can stimulate soil microbial biomass following foliar loss and thus ultimately affect belowground fauna (A'Bear et al., 2014; Bardgett & Wardle, 2003).

The negative associations between litter depth, soil microbial biomass, and earthworm biomass and density appear counterintuitive at first, as greater litter inputs are generally expected to promote surface‐feeding earthworms (Eisenhauer et al., 2009; Milcu et al., 2008; Spehn et al., 2000), and higher soil microbial biomass is often assumed to favor microbe‐feeding groups (Curry & Schmidt, 2007). However, structural equation models describe statistical associations rather than unidirectional causal effects, particularly for consumable resources such as litter and soil microbial biomass. The observed negative relationships therefore likely reflect reverse causality, whereby sites with high earthworm activity may have already consumed and incorporated litter into the soil and reduced microbial standing stocks through competition for detrital resources (Eisenhauer, 2010). This interpretation is supported by our supplementary analyses (Appendix S3: Table S1) showing that higher earthworm biomass decreased litter depth and higher earthworm density decreased soil microbial biomass, independent of roe deer and canopy condition. Given that our study was conducted 5 years after treatment establishment, it is plausible that standing stocks of litter and soil microbial biomass had already been substantially reduced by sustained earthworm activity. Future research, including pre‐treatment conditions and repeated measurements across successional stages, would allow stronger inference about how litter depth and soil microbial biomass interact with earthworms over time.

Our structural equation model results further suggest that soil water content was positively associated with total earthworm density, while our mixed‐effects model showed a positive effect of pH on total earthworm biomass. Although both soil water content and soil pH are important determinants for earthworms (de Wandeler et al., 2016; Singh et al., 2019), their relative importance differed among life stages and ecological groups in our study. Based on the structural equation models, juvenile and endogeic earthworms were positively associated with higher soil water content, while soil pH was positively related only to juvenile biomass. Roe deer presence was indirectly associated with increased soil water content, primarily through roe deer–induced changes in vegetation, particularly enhanced herb layer cover in canopy gaps. This suggests that roe deer can influence earthworms through vegetation‐mediated pathways that alter abiotic soil conditions (Ribeiro et al., 2025), with these effects being more pronounced in canopy gaps. In contrast, we found little evidence that roe deer affected soil pH, which was also reported for white‐tailed deer (Cope & Burns, 2019). In our study, soil pH was mainly explained by litter quality and likely reflects long‐term forest stand characteristics rather than short‐term roe deer effects. While browsing may influence soil pH indirectly over longer timescales through shifts in tree species composition (Pastor et al., 1988), such effects are unlikely to be detectable within the 5‐year timeframe of our study, and spatially heterogeneous nitrogen inputs from deer (Murray et al., 2013) may further obscure pH‐related patterns. Overall, roe deer appear to buffer soil moisture conditions, particularly in canopy gaps, predominantly by limiting the shrub layer, thereby benefiting earthworms. These findings provide partial support for Mechanism 5 (Figure 1).

## CONCLUSION

Earthworms are key ecosystem engineers that drive fundamental soil processes and ecosystem functioning in European temperate forests. At the same time, climate warming is expected to increase tree mortality and disturbance frequency while promoting higher roe deer densities across much of Europe. Despite the importance of these concurrent drivers, their combined effects on native earthworm communities in European forests have remained largely unexplored. Our study provides the first experimental evidence that canopy gaps and roe deer interactively influence earthworm populations in a temperate European forest, producing contrasting effects depending on forest structure. At roe deer densities of 20–30 individuals km^−2^, roe deer reduced earthworm biomass and density under closed‐canopy conditions, whereas canopy gaps increased earthworm biomass and density only when roe deer were present, primarily through changes in vegetation structure and soil water content. These results demonstrate that the effects of herbivory on belowground communities cannot be understood in isolation but instead depend critically on the disturbance context. Because our study represents a single temporal snapshot 5 years after treatment establishment, the ecological consequences of these shifts should be interpreted as context‐dependent and may differ among ecosystem functions. Reduced earthworm biomass and density under high roe deer densities in closed‐canopy forests may slow down litter incorporation and nutrient cycling, whereas higher earthworm activity in canopy gaps under roe deer presence may enhance decomposition and soil mixing (Blouin et al., 2013; Wu et al., 2025). Such short‐term belowground benefits in canopy gaps do not necessarily compensate for browsing‐related losses in tree regeneration and woody plant diversity (Lettenmaier et al., 2025). Future studies should combine repeated sampling across years with measurements of additional variables not included here, such as soil compaction, nutrient availability, and plot‐level litter quality, to clarify the mechanisms underlying deer–gap–earthworm interactions. Taken together, our results highlight that in a rapidly changing environment, ecological drivers must be studied in combination rather than isolation, as their interactive effects can fundamentally alter ecosystem responses.

## AUTHOR CONTRIBUTIONS

Ludwig Lettenmaier and Jörg Müller conceived the ideas. Sebastian Dittrich, Ludwig Lettenmaier, Giorgi Mamadashvili, Christian Ristok, and Ronja Hausmann collected the data. Ludwig Lettenmaier analyzed the data with input from Anna Bucharova, Nico Eisenhauer, Jörg Müller, and Christian Ristok. Ludwig Lettenmaier wrote the original draft. Ludwig Lettenmaier, Jörg Müller, and Christian Ristok led the writing of the manuscript, with substantial input from Nico Eisenhauer and Simone Cesarz. Christian Ammer, Anna Bucharova, Ronja Hausmann, Sebastian Dittrich, Daniel Kraus, Angelika Kölbl, Soumen Mallick, Giorgi Mamadashvili, Atle Mysterud, Kerstin Pierick, and Rike Schwarz contributed critically to the drafts and gave final approval for publication.

## CONFLICT OF INTEREST STATEMENT

The authors declare no conflicts of interest.

## Supporting information


Appendix S1.

Appendix S2.

Appendix S3.

Appendix S4.

Appendix S5.

Appendix S6.

## Data Availability

Data and code (Lettenmaier et al., 2026) are available in Zenodo at https://doi.org/10.5281/zenodo.20758912.
