# Supplementary material for: Native earthworms and roe deer in temperate forests: Friends in the sun, foes in the shade
Source: Ecology. 2026 Sep 22;107(9):e70520. doi: 10.1002/ecy.70520 (PMC13597149; doi:10.1002/ecy.70520)
Supplement: Supplementary file 1 [file ECY-107-e70520-s001.pdf]

Appendix S4

Native earthworms and roe deer in temperate forests: Friends in the sun, foes in the shade

Ludwig Lettenmaier, Nico Eisenhauer, Simone Cesarz, Christian Ammer, Anna Bucharova, Sebastian Dittrich, Ronja Hausmann, Daniel Kraus, Angelika Kölbl, Soumen Mallick, Giorgi Mamadashvili, Atle Mysterud, Kerstin Pierick, Rike Schwarz, Jörg Müller, Christian Ristok

Ecology

Piecewise structural equation models

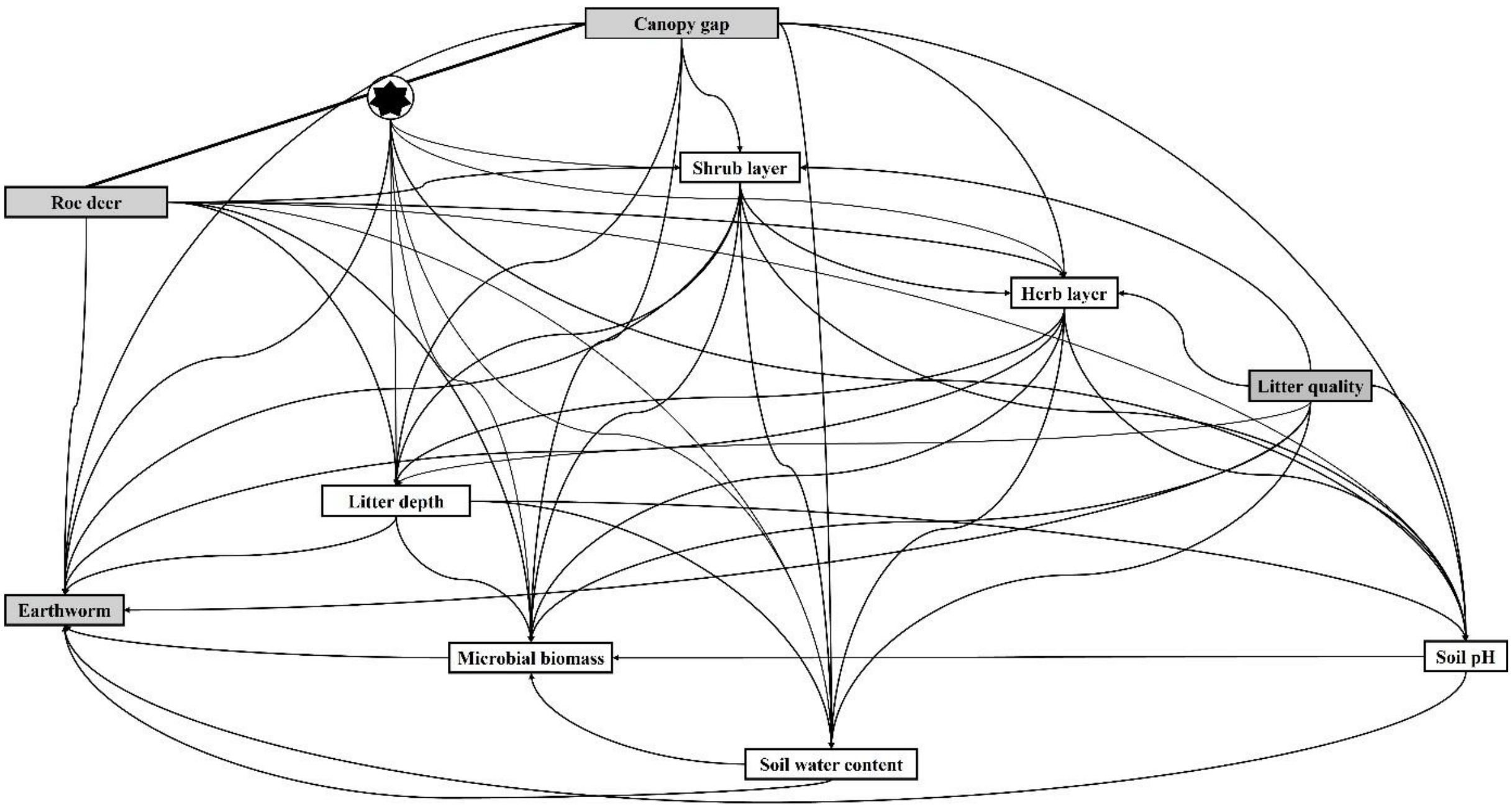

Figure S1: Conceptual path diagram underlying the piecewise structural equation models (pSEMs) testing roe deer and canopy-gap effects on earthworm communities. Boxes represent variables: roe deer treatment (absence vs. presence), canopy condition (closed gap vs. canopy gap), shrub and herb layer cover, litter depth and litter quality, soil microbial biomass, soil water content, soil pH, and earthworm responses. Single-headed arrows indicate hypothesized directional pathways evaluated in the pSEMs. We did not assign a priori positive (+) or negative (–) signs to paths because reported effects are frequently context dependent and may vary with site conditions and time since canopy gap formation. Accordingly, the pSEMs were used to explore and quantify the relative support for alternative direct and indirect pathways. Interaction paths between canopy condition and roe deer treatment were included to account for potential differences in the effects of roe deer presence versus absence under closed-canopy conditions compared with canopy gaps. Litter quality was used as a background variable for the forest patch scale (see Methods). References supporting all hypothesized paths are provided in Table S1.

Shrub and herb layer cover were modeled as responses to canopy gap, roe deer presence, their interaction, and litter quality, with shrub cover additionally included as a predictor of herb cover. Litter depth was not included in the shrub and herb layer cover models, as this would have created feedback loops, which cannot be accommodated within pSEMs (Lefcheck 2016). Litter depth was modeled as a function of canopy gap, roe deer presence, their interaction, shrub and herb layer cover, and litter quality. Soil pH and soil water content were related to canopy gap,

roe deer presence, their interaction, shrub and herb layer cover, and litter depth and quality. Residual covariance between soil pH and soil water content was included to account for shared edaphic influences. Soil microbial biomass was modeled as a function of canopy gap, roe deer presence, their interaction, shrub and herb layer cover, litter depth and quality, and soil pH and water content. Earthworm responses (biomass, density, life stages, and ecological groups) were related to canopy gap, roe deer presence, their interaction, herb and shrub layer cover, litter depth and quality, soil microbial biomass, and soil pH and water content. All mixed models included forest patch as a random effect (Figure S1).

Table S1: Conceptual justification and supporting literature for SEM paths

The table summarizes hypothesized causal pathways included in the piecewise structural equation models (pSEMs). References marked with * link the mechanism or explanatory variable directly to earthworms but not necessarily via roe deer or canopy-gap effects. (A) Roe-deer–mediated pathways are grouped by ecological mechanism to maintain clarity. (B) Interconnections among explanatory variables are shown separately. (C) Canopy-gap effects are summarized independently. (D) Litter quality paths (background variable).

A) Paths linking roe deer to earthworms

Path category	Path	Mechanism explained	Key references
**Direct roe deer effects (mechanism 1)**	Roe deer → earthworms	Roe deer directly influence soil biota through fecal deposition, altering nutrient inputs, and through trampling-induced soil compaction, which can reduce soil porosity and habitat suitability for earthworms.	Bardgett and Wardle (2003); Ramirez et al. (2020); Rearick et al. (2011)*; Jordan et al. (2000)*; McAlpine et al. (2025)*
**Vegetation-mediated indirect effects (mechanism 2)**	Roe deer → shrub layer → earthworms	Roe deer can alter shrub and herb layers cover and biomass, affecting plant nutrient demand, belowground inputs, and habitat structure, which indirectly influence earthworm populations.	Bardgett and Wardle (2003); Ramirez et al. (2020); A'Bear et al. (2014); De Wandeler et al. (2016)*
	Roe deer → herb layer → earthworms		
	Roe deer → shrub layer → herb layer → earthworms	Through selective browsing, roe deer can alter interactions between shrub and herb layers, influencing understory structure, nutrient demand, and belowground resource inputs, with indirect consequences for earthworms.	Bardgett and Wardle (2003); Beguin et al. (2011); De Wandeler et al. (2016)*
**Litter-mediated indirect effects (mechanism 3)**	Roe deer → litter depth → earthworms	Trampling and soil surface disturbance alter litter accumulation, influencing food availability and habitat conditions for earthworms.	Bardgett and Wardle (2003); Ramirez et al. (2020); De Wandeler et al. (2016)*
	Roe deer → vegetation (shrub layer/ herb layer) → litter depth → earthworms	Roe deer-induced changes in shrub and herb layers affect litterfall and ground cover, indirectly shaping litter depth available to earthworms.	
**Microbial-biomass-mediated effects (mechanism 4)**	Roe deer → soil microbial biomass → earthworms	Roe deer influence soil microbial biomass via fecal inputs. Soil microbial biomass links detrital processing to soil food webs affecting earthworms.	Bardgett and Wardle (2003); Bardgett et al. (1998); Ribeiro et al. (2025); Curry and Schmidt (2007)*
	Roe deer → vegetation (shrub layer/ herb layer) / litter depth /	Vegetation structure, litter depth, soil pH, and soil water content regulate soil microbial biomass, creating indirect pathways by which roe deer influence earthworms via soil food-web dynamics.	Bardgett and Wardle (2003); Wardle (1992); Curry and Schmidt (2007)*

Path category	Path	Mechanism explained	Key references
	soil pH / soil water content → soil microbial biomass → earthworms		
**Soil pH- and soil water content-mediated effects (mechanism 5)**	Roe deer → soil pH → earthworms	Roe deer-induced changes in nutrient inputs and vegetation can alter soil pH, which constrains earthworm survival, activity, and community composition.	Bardgett and Wardle (2003); Ramirez et al. (2020); De Wandeler et al. (2016)* Dávalos et al. (2015)*
	Roe deer → soil water content → earthworms	Roe deer-driven changes in vegetation and litter modify microclimate, infiltration, and evaporation, influencing soil water content, which affect earthworm activity and survival.	Ribeiro et al. (2025); De Wandeler et al. (2016)*; Singh et al. (2019)*; Whalen and Costa (2003)*
	Roe deer → vegetation (shrub layer/ herb layer) / litter depth→ soil pH/ soil water content→ earthworms	Vegetation- and litter-mediated effects on soil pH and soil water content create indirect pathways linking roe deer to earthworms.	Bardgett and Wardle (2003); Ramirez et al. (2020); De Wandeler et al. (2016)*; Singh et al. (2019)*; Whalen and Costa (2003)*

B) Interconnections among explanatory variables

Path category	Path	Path explained	Key references
**Vegetation structure**	Shrub layer → herb layer	Shrub layer cover influences herb layer development through competition for light, space, and nutrients.	Beguin et al. (2011)
**Vegetation → litter**	Shrub layer → litter depth	Shrub layer and herb layer cover and species composition influence litterfall rates and organic matter inputs to the soil surface.	Bressette et al. (2012); Ramirez et al. (2020)
	Herb layer → litter depth		
**Vegetation → soil chemistry**	Shrub layer → soil pH	Plant species composition and root activity influence soil pH through nutrient uptake, root exudation, litter decomposition, and rhizosphere processes.	Fu et al. (2015)
	Herb layer → soil pH		

Path category	Path	Path explained	Key references
**Vegetation → soil water content**	Shrub layer → soil water content	Shrub layer and herb layer cover modify soil water content through rainfall interception, shading (reducing evaporation), and root-driven changes in soil structure and water retention.	Zhang et al. (2024a)
	Herb layer → soil water content		
**Litter → soil chemistry**	Litter depth → soil pH	Litter accumulation and thus decomposition alters soil acidity through organic acid release and changes in the supply of exchangeable base cations.	Sayer (2006)
**Litter → soil water content**	Litter depth → soil water content	Litter accumulation enhances soil water retention by reducing evaporation, buffering erosion, and increasing infiltration, thereby slowing soil desiccation.	Sayer (2006)<br>Xu et al. (2013)
**Vegetation → microbes**	Shrub layer → soil microbial biomass	(Browsed) plants regulate nutrient demand cascading on belowground carbon inputs (roots, exudates) influencing soil microbial biomass.	Bardgett and Wardle (2003); A'Bear et al. (2014);
	Herb layer → soil microbial biomass		
**Litter → microbes**	Litter depth → soil microbial biomass	Litter accumulation provides substrate for microbial growth and activity, directly influencing soil microbial biomass.	Xu et al. (2013);<br>Berg and McClaugherty (2014)
**Soil → microbes**	Soil pH → soil microbial biomass	Soil pH and soil water content regulates microbial community composition, activity, growth and biomass.	Wardle (1992)
	Soil water content → soil microbial biomass		

C) Canopy gap effects on explanatory variables and earthworms

Path category	Path	Path explained	Key references
**Canopy → vegetation**	Canopy gap → shrub layer cover	Increased light availability promotes shrub establishment and growth following gap formation.	Zhu et al. (2014)
	Canopy gap → herb layer cover	Canopy opening increases light availability and temperature variability, promoting herb-layer development and biomass.	Kern et al. (2013, 2014)

**Path category**	**Path**	**Path explained**	**Key references**
**Canopy → litter and soil**	Canopy gap → litter depth	Forest gaps reduce litterfall due to decreased overstory biomass.	Zhang et al. (2024b)
	Canopy gap → soil microbial biomass	Canopy disturbance alters soil microclimate and substrate availability, leading to variable responses of soil microbial biomass.	Pérez-Izquierdo et al. (2025); Tong et al. (2024); Wu et al. (2019)
	Canopy gap → soil pH	Changes in litter inputs and decomposition rates following canopy opening alter soil chemical conditions, including soil pH. Reduced canopy interception and altered evapotranspiration modify soil water content dynamics in canopy gaps.	Tong et al. (2024)
	Canopy gap → soil water content		
**Direct canopy effects**	Canopy gap → earthworms	Canopy opening modifies microclimatic conditions, litter inputs, and soil physical properties, directly influencing earthworm biomass, density, and community composition.	Nachtergale et al. (2002)*; Reed et al. (2023)*

D) Litter quality paths (background variable; see Methods in the main text)

**Path category**	**Path**	**Path explained**	**Key references**
**Litter quality → vegetation**	Litter quality → shrub layer	Litter chemical traits influence soil nutrient availability and microsite conditions, affecting vegetation establishment and growth.	Xiong and Nilsson (1999)
	Litter quality → herb layer		
**Litter quality → litter depth**	Litter quality → litter depth	Differences in litter decomposability influence litter accumulation and turnover rates.	Benbow et al. (2019)
**Litter quality → microbes**	Litter quality → soil microbial biomass	Microbial responses to litter inputs depend on litter chemical traits, which regulate microbial growth.	Sayer (2006)
**Litter quality → soil chemistry**	Litter quality → soil pH	Long-term litter inputs influence soil acidity through organic acid production and base-cation release during decomposition.	Liu et al. (2009)
	Litter quality → soil water content	Litter structure and chemistry affect water retention, infiltration, and evaporation at the soil surface.	Augusto et al. (2002)

Path category	Path	Path explained	Key references
**Litter quality → earthworms**	Litter quality → earthworms	Earthworm feeding preferences, growth, and survival depend on litter chemical quality and decomposability.	De Wandeler et al. (2016)*

Results of the piecewise structural equation models

Full coefficient estimates from the piecewise structural equation models, 95% confidence intervals, test statistics, degrees of freedom, and p-values are reported in Table S2.

Canopy gaps strongly increased shrub layer cover, while roe deer presence reduced shrub cover. Shrub layer cover further increased with litter quality. Herb layer cover was negatively affected by shrub layer cover and positively influenced by litter quality. In addition, herb layer cover increased under the interaction between canopy gap and roe deer presence. Soil pH increased with litter quality but decreased with litter depth. Soil water content increased with herb layer cover and was positively affected by the interaction between canopy gap and roe deer presence. Soil microbial biomass decreased with increasing shrub layer cover, but increased with litter quality, soil pH, and soil water content.

Total earthworm biomass decreased under roe deer presence and with increasing litter depth. Total earthworm density was negatively affected by roe deer presence and soil microbial biomass but increased with soil water content. Adult earthworm biomass decreased under roe deer presence and with increasing litter depth. Adult earthworm density was also reduced by roe deer presence. Juvenile earthworm biomass increased with herb layer cover and soil pH but decreased with increasing litter quality. Juvenile earthworm density increased with herb layer cover and soil water content. Anecic earthworm biomass decreased under roe deer presence. Anecic earthworm density was negatively affected by roe deer presence and litter depth but increased under the interaction between canopy gap and roe deer presence. Endogeic earthworm biomass increased in canopy gaps, with higher herb layer cover, and with increasing soil water content, but decreased with higher soil microbial biomass. Endogeic earthworm density increased with herb layer cover and soil water content, while soil microbial biomass had a significant negative effect.

Table S2: Results of the piecewise structural equation models testing the effects of canopy gaps, roe deer presence, shrub and herb layer cover, litter depth and quality, soil pH and soil water content, and soil microbial biomass on earthworm responses (biomass, density, life stage, and ecological group).

	Response	Predictor	Estimate	95% CI lower	95% CI upper	SE	DF	Test statistic	p-value	Std. estimate	Std. 95% CI lower	Std. 95% CI upper	Std. SE
**Environmental variables**	Shrub layer	Canopy gap	1.713	1.390	2.036	0.163	150	10.484	<0.001	0.772	0.626	0.917	0.074
	Shrub layer	Roe deer presence	-0.258	-0.480	-0.037	0.112	150	-2.308	0.021	-0.130	-0.241	-0.019	0.056
	Shrub layer	Litter quality	0.142	0.031	0.254	0.056	150	2.518	0.012	0.142	0.031	0.254	0.056
	Shrub layer	Canopy gap × Roe deer presence	-0.318	-0.736	0.101	0.212	150	-1.498	0.134	-0.111	-0.256	0.035	0.074
	Herb layer	Canopy gap	-0.212	-0.833	0.409	0.314	150	-0.674	0.500	-0.095	-0.375	0.184	0.141
	Herb layer	Roe deer presence	-0.068	-0.338	0.203	0.137	150	-0.495	0.621	-0.034	-0.170	0.102	0.069
	Herb layer	Shrub layer	-0.228	-0.458	0.002	0.116	150	-1.960	0.050	-0.228	-0.458	0.002	0.116
	Herb layer	Litter quality	0.202	0.001	0.404	0.102	150	1.982	0.048	0.202	0.001	0.404	0.102
	Herb layer	Canopy gap × Roe deer presence	0.532	0.024	1.041	0.257	150	2.069	0.039	0.185	0.008	0.362	0.090
	Litter depth	Canopy gap	-0.507	-1.137	0.123	0.319	150	-1.589	0.112	-0.228	-0.512	0.056	0.144
	Litter depth	Roe deer presence	-0.217	-0.520	0.086	0.153	150	-1.413	0.158	-0.109	-0.261	0.043	0.077
	Litter depth	Shrub layer	-0.143	-0.384	0.099	0.122	150	-1.169	0.243	-0.143	-0.384	0.099	0.122
	Litter depth	Herb layer	-0.054	-0.219	0.111	0.084	150	-0.646	0.518	-0.054	-0.219	0.111	0.084
	Litter depth	Litter quality	0.110	-0.078	0.299	0.096	150	1.155	0.248	0.110	-0.078	0.299	0.096
	Litter depth	Canopy gap × Roe deer presence	-0.139	-0.713	0.435	0.290	150	-0.479	0.632	-0.048	-0.248	0.151	0.101
	Soil pH	Canopy gap	-0.185	-0.716	0.346	0.269	150	-0.687	0.492	-0.083	-0.322	0.156	0.121
	Soil pH	Roe deer presence	0.058	-0.164	0.280	0.112	150	0.515	0.606	0.029	-0.082	0.140	0.056
	Soil pH	Shrub layer	0.124	-0.068	0.315	0.097	150	1.275	0.202	0.124	-0.068	0.315	0.097
	Soil pH	Herb layer	0.082	-0.058	0.222	0.071	150	1.156	0.248	0.082	-0.058	0.222	0.071
	Soil pH	Litter quality	0.478	0.295	0.661	0.093	150	5.155	<0.001	0.478	0.295	0.661	0.093
	Soil pH	Litter depth	-0.160	-0.294	-0.026	0.068	150	-2.368	0.018	-0.160	-0.294	-0.026	0.068
	Soil pH	Canopy gap × Roe deer presence	-0.226	-0.646	0.194	0.213	150	-1.063	0.288	-0.079	-0.225	0.068	0.074
	Soil water content	Canopy gap	-0.118	-0.732	0.497	0.311	150	-0.378	0.705	-0.053	-0.330	0.224	0.14
	Soil water content	Roe deer presence	-0.189	-0.488	0.110	0.151	150	-1.246	0.213	-0.095	-0.245	0.055	0.076
	Soil water content	Shrub layer	0.229	-0.006	0.464	0.119	150	1.929	0.054	0.229	-0.006	0.464	0.119

	Response	Predictor	Estimate	95% CI lower	95% CI upper	SE	DF	Test statistic	p-value	Std. estimate	Std. 95% CI lower	Std. 95% CI upper	Std. SE
	Soil water content	Herb layer	0.237	0.079	0.395	0.08	150	2.958	0.003	0.237	0.079	0.395	0.080
	Soil water content	Litter quality	0.065	-0.116	0.247	0.092	150	0.712	0.477	0.065	-0.116	0.247	0.092
	Soil water content	Litter depth	-0.037	-0.194	0.120	0.079	150	-0.468	0.640	-0.037	-0.194	0.120	0.079
	Soil water content	Canopy gap × Roe deer presence	0.717	0.153	1.280	0.285	150	2.514	0.012	0.250	0.053	0.446	0.099
	Soil microbial biomass	Canopy gap	-0.467	-0.974	0.041	0.257	150	-1.818	0.069	-0.210	-0.439	0.018	0.116
	Soil microbial biomass	Roe deer presence	-0.117	-0.371	0.137	0.129	150	-0.907	0.364	-0.059	-0.186	0.069	0.064
	Soil microbial biomass	Shrub layer	-0.219	-0.416	-0.021	0.100	150	-2.184	0.029	-0.219	-0.416	-0.021	0.100
	Soil microbial biomass	Herb layer	0.022	-0.113	0.156	0.068	150	0.320	0.749	0.022	-0.113	0.156	0.068
	Soil microbial biomass	Litter quality	0.187	0.026	0.349	0.082	150	2.289	0.022	0.187	0.026	0.349	0.082
	Soil microbial biomass	Litter depth	0.104	-0.027	0.235	0.066	150	1.566	0.117	0.104	-0.027	0.235	0.066
	Soil microbial biomass	Soil pH	0.296	0.136	0.456	0.081	150	3.657	<0.001	0.296	0.136	0.456	0.081
	Soil microbial biomass	Soil water content	0.398	0.254	0.543	0.073	150	5.450	<0.001	0.398	0.254	0.543	0.073
	Soil microbial biomass	Canopy gap × Roe deer presence	-0.244	-0.735	0.247	0.248	150	-0.983	0.326	-0.085	-0.256	0.086	0.086
	~~Soil pH	~~Soil water content	0.418				150	5.574	<0.001	0.418			
Total earthworms	Biomass	Canopy gap	-0.116	-0.769	0.537	0.330	150	-0.351	0.726	-0.052	-0.346	0.242	0.149
	Biomass	Roe deer presence	-0.453	-0.798	-0.108	0.174	150	-2.597	0.009	-0.227	-0.400	-0.054	0.087
	Biomass	Shrub layer	-0.003	-0.260	0.255	0.130	150	-0.021	0.983	-0.003	-0.260	0.255	0.130
	Biomass	Herb layer	0.087	-0.079	0.253	0.084	150	1.039	0.299	0.087	-0.079	0.253	0.084
	Biomass	Soil microbial biomass	-0.062	-0.267	0.144	0.104	150	-0.594	0.552	-0.062	-0.267	0.144	0.104
	Biomass	Litter depth	-0.171	-0.338	-0.003	0.085	150	-2.017	0.044	-0.171	-0.338	-0.003	0.085
	Biomass	Litter quality	-0.024	-0.223	0.175	0.101	150	-0.236	0.813	-0.024	-0.223	0.175	0.101
	Biomass	Soil pH	0.176	-0.033	0.384	0.106	150	1.665	0.096	0.176	-0.033	0.384	0.106
	Biomass	Soil water content	0.080	-0.120	0.281	0.101	150	0.793	0.428	0.080	-0.120	0.281	0.101
	Biomass	Canopy gap × Roe deer presence	0.493	-0.170	1.156	0.336	150	1.468	0.142	0.172	-0.059	0.403	0.117
	Density	Canopy gap	0.282	-0.316	0.879	0.302	150	0.932	0.352	0.127	-0.142	0.396	0.136
	Density	Roe deer presence	-0.411	-0.727	-0.096	0.160	150	-2.574	0.010	-0.206	-0.364	-0.048	0.080
	Density	Shrub layer	-0.193	-0.429	0.043	0.119	150	-1.615	0.106	-0.193	-0.429	0.043	0.119
	Density	Herb layer	0.139	-0.015	0.292	0.078	150	1.788	0.074	0.139	-0.015	0.292	0.078
	Density	Soil microbial biomass	-0.223	-0.411	-0.034	0.095	150	-2.336	0.020	-0.223	-0.411	-0.034	0.095

	Response	Predictor	Estimate	95% CI lower	95% CI upper	SE	DF	Test statistic	p-value	Std. estimate	Std. 95% CI lower	Std. 95% CI upper	Std. SE
	Density	Litter depth	-0.143	-0.296	0.011	0.078	150	-1.833	0.067	-0.143	-0.296	0.011	0.078
	Density	Litter quality	0.081	-0.102	0.264	0.093	150	0.872	0.383	0.081	-0.102	0.264	0.093
	Density	Soil pH	0.149	-0.042	0.340	0.096	150	1.544	0.123	0.149	-0.042	0.340	0.096
	Density	Soil water content	0.244	0.061	0.427	0.092	150	2.636	0.008	0.244	0.061	0.427	0.092
	Density	Canopy gap × Roe deer presence	0.558	-0.049	1.165	0.307	150	1.818	0.069	0.194	-0.017	0.406	0.107
**Life stage**	Adult biomass	Canopy gap	-0.250	-0.916	0.417	0.337	150	-0.740	0.459	-0.112	-0.413	0.188	0.152
	Adult biomass	Roe deer presence	-0.390	-0.736	-0.044	0.175	150	-2.227	0.026	-0.196	-0.369	-0.022	0.088
	Adult biomass	Shrub layer	-0.019	-0.280	0.242	0.132	150	-0.143	0.886	-0.019	-0.280	0.242	0.132
	Adult biomass	Herb layer	-0.008	-0.178	0.162	0.086	150	-0.095	0.924	-0.008	-0.178	0.162	0.086
	Adult biomass	Soil microbial biomass	-0.093	-0.303	0.118	0.106	150	-0.870	0.384	-0.093	-0.303	0.118	0.106
	Adult biomass	Litter depth	-0.202	-0.374	-0.031	0.087	150	-2.328	0.020	-0.202	-0.374	-0.031	0.087
	Adult biomass	Litter quality	0.115	-0.092	0.321	0.104	150	1.098	0.272	0.115	-0.092	0.321	0.104
	Adult biomass	Soil pH	0.069	-0.150	0.288	0.111	150	0.624	0.533	0.069	-0.150	0.288	0.111
	Adult biomass	Soil water content	0.133	-0.072	0.339	0.104	150	1.282	0.200	0.133	-0.072	0.339	0.104
	Adult biomass	Canopy gap × Roe deer presence	0.271	-0.396	0.939	0.338	150	0.804	0.422	0.094	-0.138	0.327	0.118
	Adult density	Canopy gap	-0.04	-0.713	0.633	0.341	150	-0.119	0.905	-0.018	-0.321	0.285	0.153
	Adult density	Roe deer presence	-0.482	-0.853	-0.111	0.188	150	-2.563	0.010	-0.242	-0.428	-0.055	0.094
	Adult density	Shrub layer	-0.109	-0.373	0.154	0.134	150	-0.820	0.412	-0.109	-0.373	0.154	0.134
	Adult density	Herb layer	-0.057	-0.226	0.112	0.086	150	-0.669	0.504	-0.057	-0.226	0.112	0.086
	Adult density	Soil microbial biomass	-0.190	-0.401	0.021	0.107	150	-1.784	0.074	-0.190	-0.401	0.021	0.107
	Adult density	Litter depth	-0.002	-0.173	0.170	0.087	150	-0.021	0.983	-0.002	-0.173	0.170	0.087
	Adult density	Litter quality	0.151	-0.047	0.350	0.100	150	1.508	0.132	0.151	-0.047	0.350	0.100
	Adult density	Soil pH	0.093	-0.117	0.303	0.106	150	0.875	0.382	0.093	-0.117	0.303	0.106
	Adult density	Soil water content	0.130	-0.072	0.333	0.102	150	1.273	0.203	0.130	-0.072	0.333	0.102
	Adult density	Canopy gap × Roe deer presence	0.279	-0.433	0.991	0.360	150	0.774	0.439	0.097	-0.151	0.345	0.125
	Juvenile biomass	Canopy gap	0.132	-0.524	0.787	0.332	150	0.396	0.692	0.059	-0.236	0.354	0.149
	Juvenile biomass	Roe deer presence	-0.306	-0.668	0.055	0.183	150	-1.673	0.094	-0.154	-0.335	0.028	0.092
	Juvenile biomass	Shrub layer	0.047	-0.210	0.304	0.130	150	0.364	0.716	0.047	-0.210	0.304	0.130
	Juvenile biomass	Herb layer	0.194	0.030	0.359	0.083	150	2.332	0.020	0.194	0.030	0.359	0.083

	Response	Predictor	Estimate	95% CI lower	95% CI upper	SE	DF	Test statistic	p-value	Std. estimate	Std. 95% CI lower	Std. 95% CI upper	Std. SE
	Juvenile biomass	Soil microbial biomass	0.020	-0.186	0.225	0.104	150	0.188	0.851	0.020	-0.186	0.225	0.104
	Juvenile biomass	Litter depth	-0.053	-0.220	0.114	0.085	150	-0.628	0.530	-0.053	-0.220	0.114	0.085
	Juvenile biomass	Litter quality	-0.211	-0.404	-0.018	0.098	150	-2.156	0.031	-0.211	-0.404	-0.018	0.098
	Juvenile biomass	Soil pH	0.205	0.000	0.410	0.104	150	1.978	0.048	0.205	0.000	0.410	0.104
	Juvenile biomass	Soil water content	-0.022	-0.219	0.175	0.100	150	-0.218	0.827	-0.022	-0.219	0.175	0.100
	Juvenile biomass	Canopy gap × Roe deer presence	0.525	-0.169	1.219	0.351	150	1.495	0.135	0.183	-0.059	0.424	0.122
	Juvenile density	Canopy gap	0.318	-0.280	0.915	0.302	150	1.050	0.294	0.143	-0.126	0.412	0.136
	Juvenile density	Roe deer presence	-0.303	-0.615	0.010	0.158	150	-1.915	0.056	-0.152	-0.308	0.005	0.079
	Juvenile density	Shrub layer	-0.178	-0.414	0.057	0.119	150	-1.496	0.134	-0.178	-0.414	0.057	0.119
	Juvenile density	Herb layer	0.168	0.014	0.323	0.078	150	2.152	0.031	0.168	0.014	0.323	0.078
	Juvenile density	Soil microbial biomass	-0.183	-0.372	0.005	0.096	150	-1.918	0.055	-0.183	-0.372	0.005	0.096
	Juvenile density	Litter depth	-0.153	-0.308	0.002	0.078	150	-1.953	0.051	-0.153	-0.308	0.002	0.078
	Juvenile density	Litter quality	0.040	-0.145	0.225	0.094	150	0.427	0.669	0.040	-0.145	0.225	0.094
	Juvenile density	Soil pH	0.140	-0.052	0.331	0.097	150	1.443	0.149	0.140	-0.052	0.331	0.097
	Juvenile density	Soil water content	0.227	0.044	0.410	0.093	150	2.454	0.014	0.227	0.044	0.410	0.093
	Juvenile density	Canopy gap × Roe deer presence	0.537	-0.065	1.139	0.304	150	1.764	0.078	0.187	-0.022	0.396	0.106
**Ecological group**	Anecic biomass	Canopy gap	-0.331	-0.994	0.332	0.336	150	-0.988	0.323	-0.149	-0.448	0.149	0.151
	Anecic biomass	Roe deer presence	-0.418	-0.761	-0.075	0.174	150	-2.405	0.016	-0.210	-0.382	-0.037	0.087
	Anecic biomass	Shrub layer	0.046	-0.215	0.306	0.132	150	0.345	0.730	0.046	-0.215	0.306	0.132
	Anecic biomass	Herb layer	0.049	-0.121	0.219	0.086	150	0.571	0.568	0.049	-0.121	0.219	0.086
	Anecic biomass	Soil microbial biomass	-0.001	-0.210	0.208	0.106	150	-0.014	0.989	-0.001	-0.210	0.208	0.106
	Anecic biomass	Litter depth	-0.163	-0.334	0.007	0.086	150	-1.893	0.058	-0.163	-0.334	0.007	0.086
	Anecic biomass	Litter quality	-0.051	-0.256	0.154	0.104	150	-0.491	0.624	-0.051	-0.256	0.154	0.104
	Anecic biomass	Soil pH	0.170	-0.043	0.383	0.108	150	1.581	0.114	0.170	-0.043	0.383	0.108
	Anecic biomass	Soil water content	0.044	-0.160	0.247	0.103	150	0.423	0.672	0.044	-0.160	0.247	0.103
	Anecic biomass	Canopy gap × Roe deer presence	0.620	-0.042	1.281	0.335	150	1.852	0.064	0.216	-0.014	0.446	0.117
	Anecic density	Canopy gap	0.010	-0.648	0.668	0.333	150	0.030	0.976	0.004	-0.295	0.304	0.151
	Anecic density	Roe deer presence	-0.472	-0.802	-0.142	0.167	150	-2.827	0.005	-0.237	-0.402	-0.071	0.084
	Anecic density	Shrub layer	-0.174	-0.437	0.090	0.133	150	-1.304	0.192	-0.174	-0.437	0.09	0.133

Response	Predictor	Estimate	95% CI lower	95% CI upper	SE	DF	Test statistic	p-value	Std. estimate	Std. 95% CI lower	Std. 95% CI upper	Std. SE
Anecic density	Herb layer	-0.033	-0.207	0.141	0.088	150	-0.373	0.709	-0.033	-0.207	0.141	0.088
Anecic density	Soil microbial biomass	0.031	-0.176	0.239	0.105	150	0.297	0.766	0.031	-0.176	0.239	0.105
Anecic density	Litter depth	-0.206	-0.376	-0.036	0.086	150	-2.393	0.017	-0.206	-0.376	-0.036	0.086
Anecic density	Litter quality	0.031	-0.180	0.242	0.107	150	0.290	0.772	0.031	-0.180	0.242	0.107
Anecic density	Soil pH	0.074	-0.141	0.288	0.108	150	0.678	0.498	0.074	-0.141	0.288	0.108
Anecic density	Soil water content	0.090	-0.115	0.295	0.104	150	0.869	0.385	0.090	-0.115	0.295	0.104
Anecic density	Canopy gap × Roe deer presence	0.649	0.012	1.286	0.322	150	2.013	0.044	0.226	0.004	0.448	0.112
Endogeic biomass	Canopy gap	0.808	0.185	1.432	0.316	150	2.563	0.010	0.364	0.083	0.645	0.142
Endogeic biomass	Roe deer presence	-0.236	-0.573	0.101	0.171	150	-1.382	0.167	-0.118	-0.288	0.051	0.086
Endogeic biomass	Shrub layer	-0.211	-0.457	0.035	0.124	150	-1.692	0.091	-0.211	-0.457	0.035	0.124
Endogeic biomass	Herb layer	0.180	0.021	0.338	0.080	150	2.234	0.025	0.180	0.021	0.338	0.080
Endogeic biomass	Soil microbial biomass	-0.287	-0.488	-0.087	0.101	150	-2.837	0.005	-0.287	-0.488	-0.087	0.101
Endogeic biomass	Litter depth	-0.054	-0.215	0.107	0.081	150	-0.665	0.506	-0.054	-0.215	0.107	0.081
Endogeic biomass	Litter quality	0.127	-0.062	0.315	0.095	150	1.328	0.184	0.127	-0.062	0.315	0.095
Endogeic biomass	Soil pH	0.062	-0.136	0.259	0.100	150	0.618	0.537	0.062	-0.136	0.259	0.100
Endogeic biomass	Soil water content	0.237	0.046	0.428	0.097	150	2.447	0.014	0.237	0.046	0.428	0.097
Endogeic biomass	Canopy gap × Roe deer presence	-0.379	-1.027	0.268	0.328	150	-1.158	0.247	-0.132	-0.358	0.093	0.114
Endogeic density	Canopy gap	0.366	-0.211	0.944	0.292	150	1.253	0.210	0.165	-0.095	0.425	0.132
Endogeic density	Roe deer presence	-0.230	-0.517	0.057	0.145	150	-1.581	0.114	-0.115	-0.260	0.029	0.073
Endogeic density	Shrub layer	-0.170	-0.395	0.055	0.114	150	-1.490	0.136	-0.170	-0.395	0.055	0.114
Endogeic density	Herb layer	0.206	0.057	0.355	0.076	150	2.726	0.006	0.206	0.057	0.355	0.076
Endogeic density	Soil microbial biomass	-0.348	-0.531	-0.166	0.092	150	-3.773	<0.001	-0.348	-0.531	-0.166	0.092
Endogeic density	Litter depth	-0.029	-0.179	0.121	0.076	150	-0.382	0.702	-0.029	-0.179	0.121	0.076
Endogeic density	Litter quality	0.112	-0.075	0.298	0.094	150	1.183	0.237	0.112	-0.075	0.298	0.094
Endogeic density	Soil pH	0.147	-0.042	0.336	0.096	150	1.535	0.125	0.147	-0.042	0.336	0.096
Endogeic density	Soil water content	0.311	0.133	0.489	0.090	150	3.451	0.001	0.311	0.133	0.489	0.090
Endogeic density	Canopy gap × Roe deer presence	0.342	-0.214	0.897	0.281	150	1.216	0.224	0.119	-0.074	0.312	0.098

Notes: Response indicates the dependent variable of each component model, and Predictor indicates the corresponding explanatory variable. Estimate denotes the unstandardized regression coefficient, and Std. estimate denotes the standardized path coefficient. CI denotes 95% confidence interval,

SE denotes standard error, and DF denotes degrees of freedom. Test statistic denotes the critical value returned by piecewiseSEM::coefs() and is calculated as the coefficient estimate divided by its standard error. Confidence intervals for unstandardized coefficients were calculated from coefficient estimates and standard errors using the corresponding critical value based on the reported degrees of freedom. Approximate confidence intervals for standardized coefficients were calculated by applying the same standardization factor to the standard errors. Confidence intervals are not shown for correlated-error terms where no standard error was returned.

Calculation of direct, indirect, and net effects (piecewise structural equation models path decomposition)

To quantify how roe deer presence and canopy gaps influenced earthworm responses, we decomposed effects derived from the piecewise structural equation models (pSEMs) into direct, indirect, and net components using standardized path coefficients (Table S3). Standardized coefficients were extracted from all component models of the pSEM. Effect decomposition was based on the set of directional relationships specified in the pSEM, linking experimental treatments (roe deer presence/absence and canopy gap/closed canopy), mediating variables (vegetation cover, litter depth, soil microbial biomass, soil pH, and soil water content), and the focal earthworm response. Each modeled relationship corresponds to a path from a predictor to a response variable, weighted by its standardized coefficient. Where component models included Canopy × Roe deer interaction terms, canopy-specific roe deer effects were derived using a simple-slope approximation based on standardized coefficients. For each response variable affected by an interaction, the conditional standardized effect of roe deer browsing was calculated as:

$$\beta_{\text{Roe deer}\to Y|\text{canopy gap}} = \beta_{\text{Roe deer}\to Y} + \beta_{\text{Roe deer}\times\text{Canopy gap}\to Y} \cdot \text{Canopy gap}$$

where Canopy gap = 0 represents closed canopy and Canopy gap = 1 represents canopy gap. Interaction terms were not treated as separate paths but were incorporated through these canopy-specific coefficients, allowing effect decomposition to be conducted separately for closed-canopy and canopy-gap conditions. Direct effects were defined as the standardized path coefficient linking a predictor (roe deer presence or canopy gap) directly to an earthworm response. Indirect effects were quantified by identifying all pSEM paths from a predictor to the earthworm response that included one or more mediating variables. For each indirect pathway, the contribution was calculated as the product of standardized coefficients along that path. Total indirect effects were obtained by summing contributions across all indirect pathways. Net effects were calculated as the sum of direct and total indirect effects, providing an integrated measure of the overall association between a predictor and an earthworm response implied by the pSEM.

Table S3: Decomposition of standardized effects of roe deer presence on earthworm biomass and density across canopy conditions.

**Response**	**Canopy**	**Direct**	**Indirect**	**Net**
Total biomass	Closed canopy	-0.227	0.015	-0.212
	Canopy gap	-0.056	0.050	-0.006
Total density	Closed canopy	-0.206	0.029	-0.177
	Canopy gap	-0.012	0.137	0.125
Juvenile biomass	Closed canopy	-0.154	0.004	-0.149
	Canopy gap	0.029	0.023	0.052
Juvenile density	Closed canopy	-0.152	0.027	-0.125
	Canopy gap	0.035	0.137	0.172
Adult biomass	Closed canopy	-0.196	0.014	-0.182
	Canopy gap	-0.101	0.052	-0.049
Adult density	Closed canopy	-0.242	0.016	-0.225
	Canopy gap	-0.145	0.041	-0.104
Anecic biomass	Closed canopy	-0.210	0.008	-0.202
	Canopy gap	0.006	0.021	0.027
Anecic density	Closed canopy	-0.237	0.030	-0.207
	Canopy gap	-0.011	0.072	0.061
Endogeic biomass	Closed canopy	-0.118	0.027	-0.091
	Canopy gap	-0.250	0.144	-0.107
Endogeic density	Closed canopy	-0.115	0.018	-0.098
	Canopy gap	0.004	0.147	0.150

Notes: Shown are standardized direct effects, indirect effects, and net effects of roe deer presence derived from the piecewise structural equation models. Direct effects represent standardized paths from roe deer presence to the response. Indirect effects represent the summed products of standardized path coefficients through vegetation structure, litter depth, soil microbial biomass, soil pH, and soil water content. Net effects are the sum of direct and indirect effects.

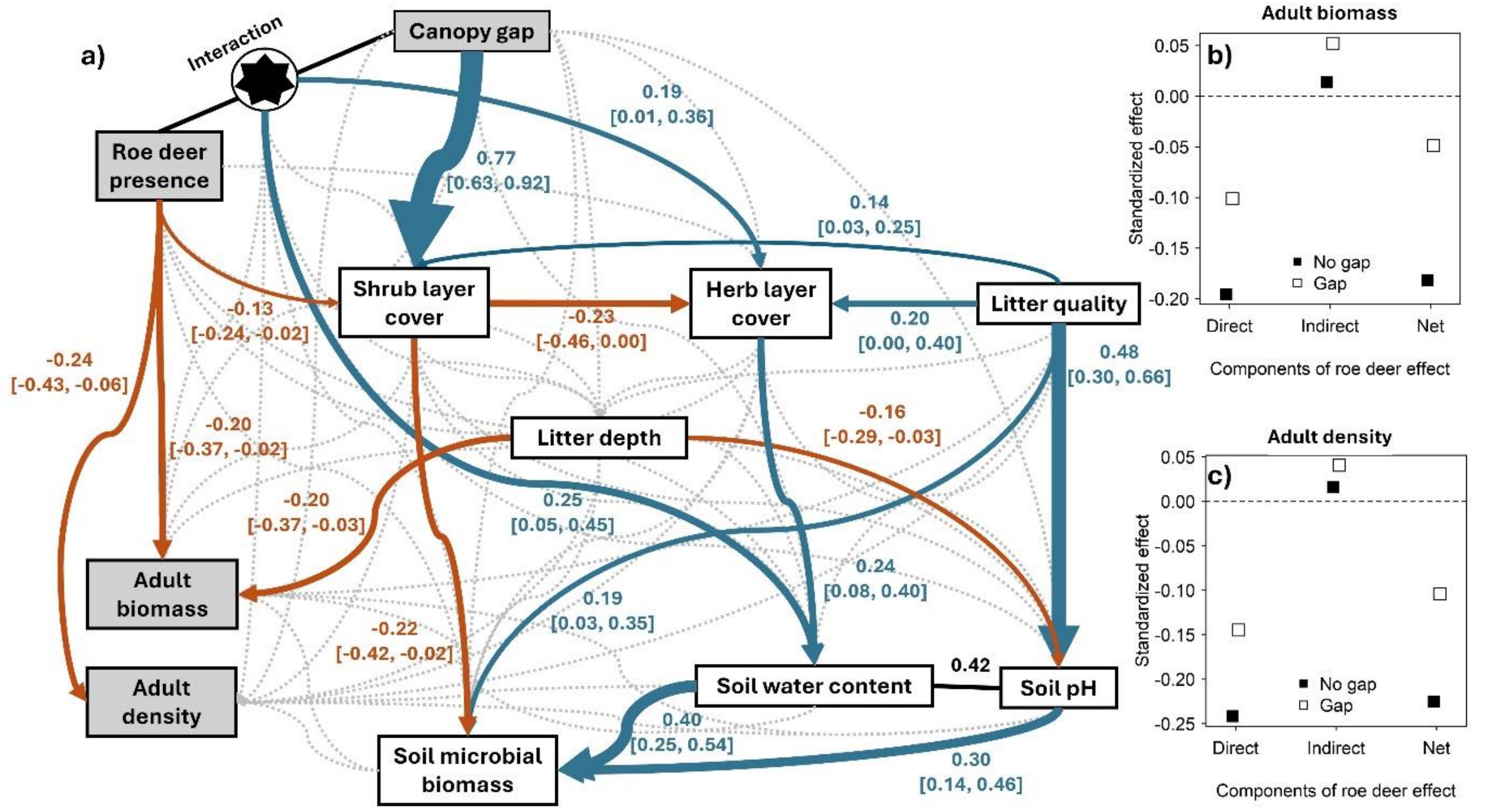

Figure S2: (a) Piecewise structural equation model (pSEM) illustrating pathways linking canopy gaps, roe deer presence, shrub and herb layer cover, litter depth and quality (C:N ratio), soil pH, soil water content, and soil microbial biomass to adult earthworm biomass and density. Arrow labels show standardized path coefficients with approximate 95% confidence intervals in brackets. Paths are color-coded by effect direction (blue = significant positive, orange = significant negative, grey dashed = non-significant). The black line indicates covariance among soil pH and soil water content, and the black line with a star denotes the canopy × roe deer interaction. Arrow thickness is proportional to standardized path coefficients. Standardized direct, indirect, and net effects of roe deer presence on adult earthworm **(b)** biomass and **(c)** density are derived from the pSEMs. Filled symbols represent closed canopy patches, and open symbols represent patches with canopy gaps. Net effects equal the sum of standardized direct and indirect effects. Direct, indirect, and net effects are based on all modelled paths, including non-significant ones. Full unstandardized and standardized pSEM coefficients, including 95% confidence intervals, test statistics, degrees of freedom, and p-values, are reported in Table S2.

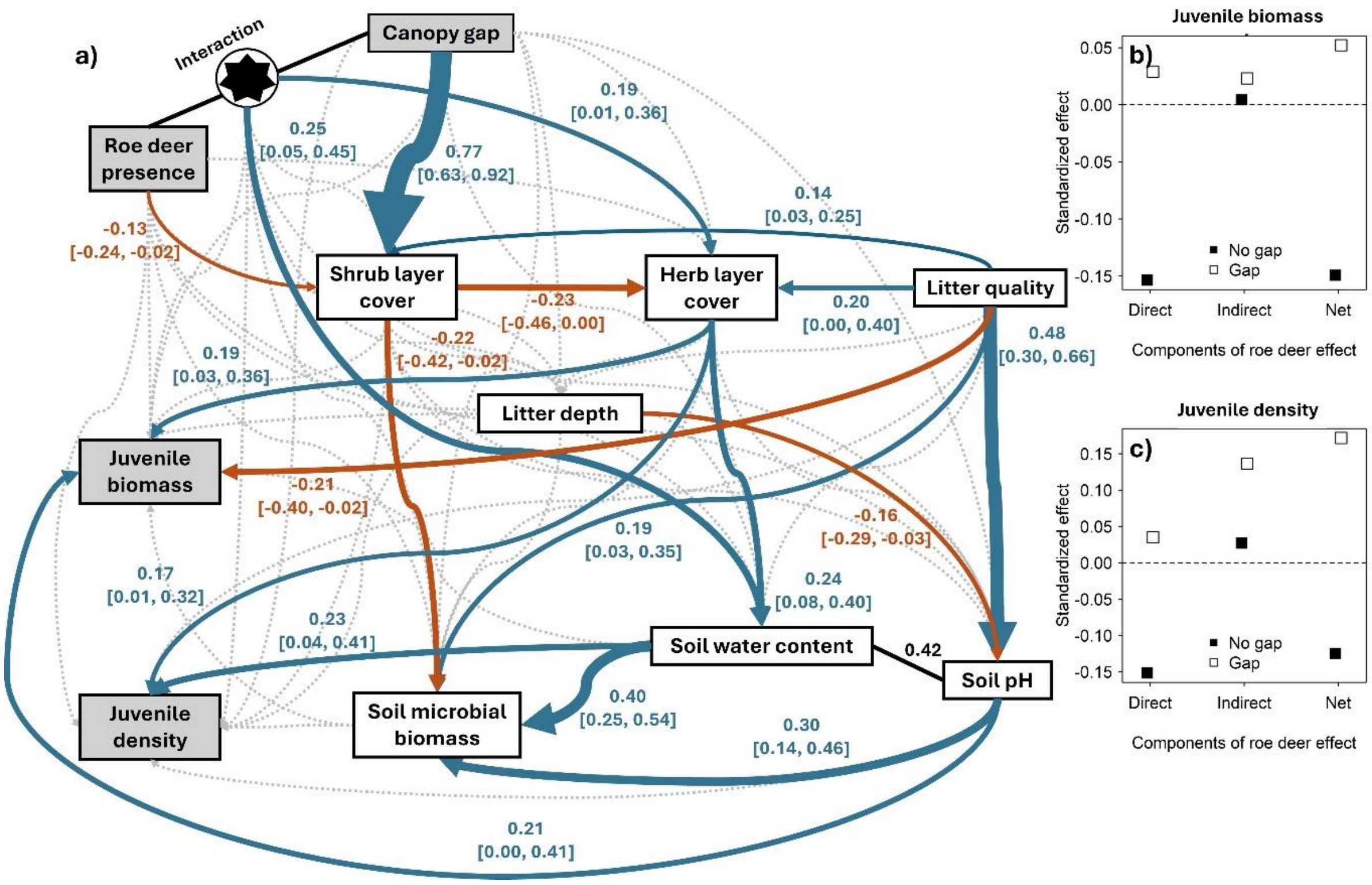

Figure S3: **(a)** Piecewise structural equation model (pSEM) illustrating pathways linking canopy gaps, roe deer presence, shrub and herb layer cover, litter depth and quality (C:N ratio), soil pH, soil water content, and soil microbial biomass to juvenile earthworm biomass and density. Arrow labels show standardized path coefficients with approximate 95% confidence intervals in brackets. Paths are color-coded by effect direction (blue = significant positive, orange = significant negative, grey dashed = non-significant). The black line indicates covariance among soil pH and soil water content, and the black line with a star denotes the canopy × roe deer interaction. Arrow thickness is proportional to standardized path coefficients. Standardized direct, indirect, and net effects of roe deer presence on juvenile earthworm **(b)** biomass and **(c)** density are derived from the pSEMs. Filled symbols represent closed canopy patches, and open symbols represent patches with canopy gaps. Net effects equal the sum of standardized direct and indirect effects. Direct, indirect, and net effects are based on all modelled paths, including non-significant ones. Full unstandardized and standardized pSEM coefficients, including 95% confidence intervals, test statistics, degrees of freedom, and p-values, are reported in Table S2.

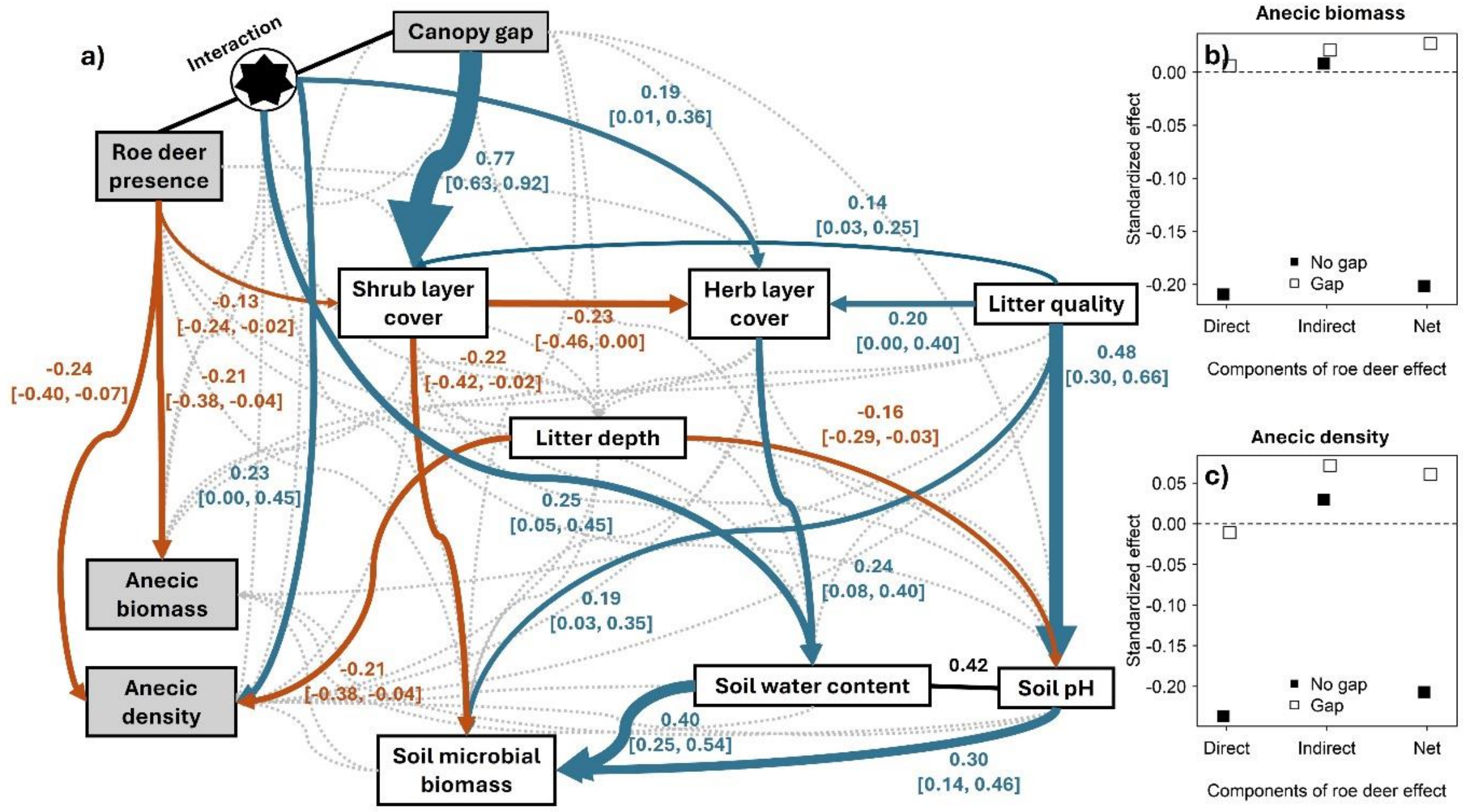

Figure S4: (a) Piecewise structural equation model (pSEM) illustrating pathways linking canopy gaps, roe deer presence, shrub and herb layer cover, litter depth and quality (C:N ratio), soil pH, soil water content, and soil microbial biomass to anecic earthworm biomass and density. Arrow labels show standardized path coefficients with approximate 95% confidence intervals in brackets. Paths are color-coded by effect direction (blue = significant positive, orange = significant negative, grey dashed = non-significant). The black line indicates covariance among soil pH and soil water content, and the black line with a star denotes the canopy × roe deer interaction. Arrow thickness is proportional to standardized path coefficients. Standardized direct, indirect, and net effects of roe deer presence on anecic earthworm **(b)** biomass and **(c)** density are derived from the pSEMs. Filled symbols represent closed canopy patches, and open symbols represent patches with canopy gaps. Net effects equal the sum of standardized direct and indirect effects. Direct, indirect, and net effects are based on all modelled paths, including non-significant ones. Full unstandardized and standardized pSEM coefficients, including 95% confidence intervals, test statistics, degrees of freedom, and p-values, are reported in Table S2.

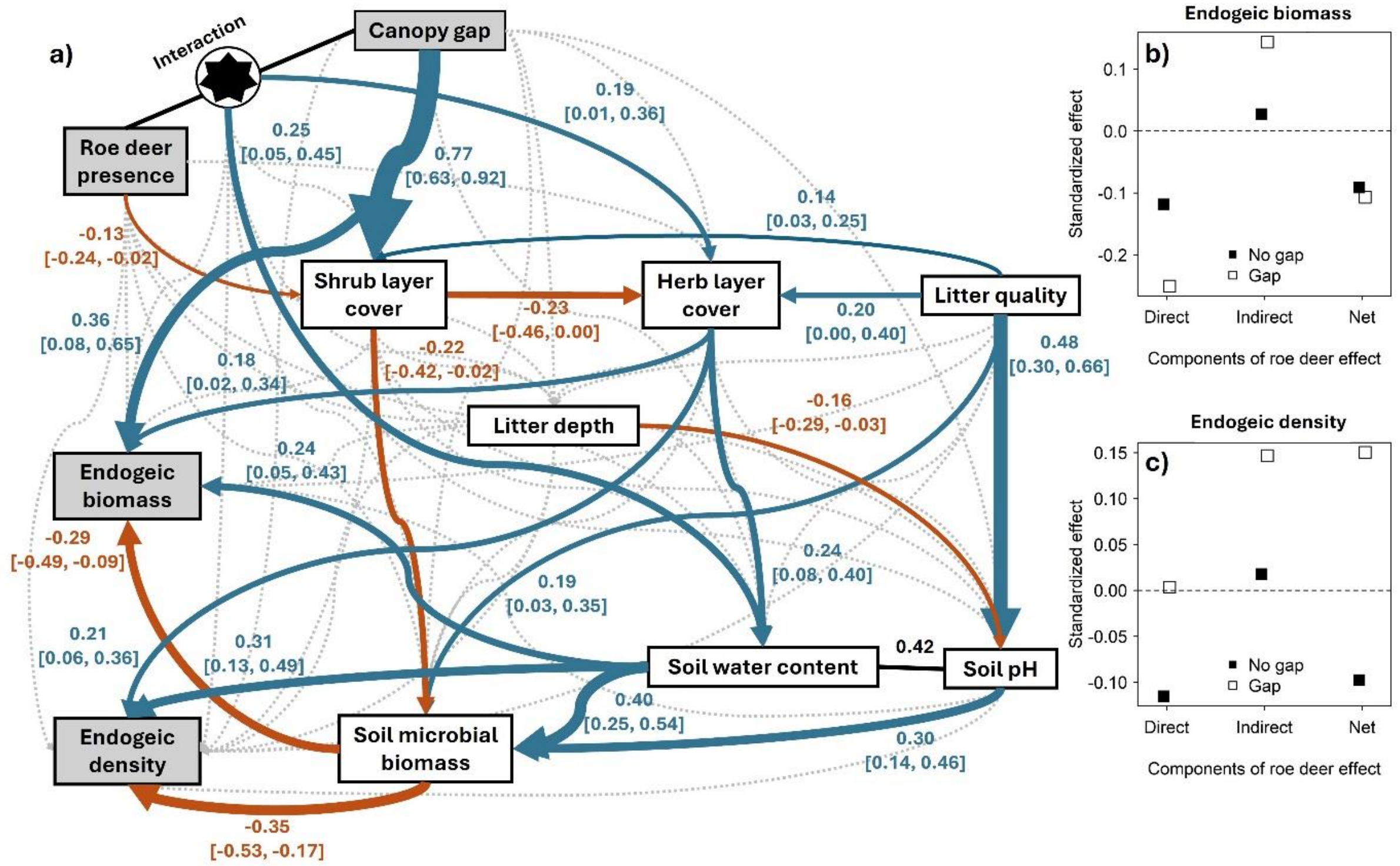

Figure S5: (a) Piecewise structural equation model (pSEM) illustrating pathways linking canopy gaps, roe deer presence, shrub and herb layer cover, litter depth and quality (C:N ratio), soil pH, soil water content, and soil microbial biomass to endogeic earthworm biomass and density. Arrow labels show standardized path coefficients with approximate 95% confidence intervals in brackets. Paths are color-coded by effect direction (blue = significant positive, orange = significant negative, grey dashed = non-significant). The black line indicates covariance among soil pH and soil water content, and the black line with a star denotes the canopy × roe deer interaction. Arrow thickness is proportional to standardized path coefficients. Standardized direct, indirect, and net effects of roe deer presence on endogeic earthworm **(b)** biomass and **(c)** density are derived from the pSEMs. Filled symbols represent closed canopy patches, and open symbols represent patches with canopy gaps. Net effects equal the sum of standardized direct and indirect effects. Direct, indirect, and net effects are based on all modelled paths, including non-significant ones. Full unstandardized and standardized pSEM coefficients, including 95% confidence intervals, test statistics, degrees of freedom, and p-values, are reported in Table S2.

References

A'Bear, A. D., S. N. Johnson, and T. H. Jones. 2014. Putting the 'upstairs–downstairs' into ecosystem service: What can aboveground–belowground ecology tell us? Biological Control 75:97–107.

Augusto, L., J. Ranger, D. Binkley, and A. Rothe. 2002. Impact of several common tree species of European temperate forests on soil fertility. Annals of Forest Science 59:233–253.

Bardgett, R. D., and D. A. Wardle. 2003. Herbivore-mediated linkages between aboveground and belwoground communities. Ecology 84:2258–2268.

Bardgett, R. D., D. A. Wardle, and G. W. Yeates. 1998. Linking above-ground and below-ground interactions: how plant responses to foliar herbivory influence soil organisms. Soil Biology and Biochemistry 30:1867–1878.

Beguin, J., D. Pothier, and S. D. Côté. 2011. Deer browsing and soil disturbance induce cascading effects on plant communities: a multilevel path analysis. Ecological Applications 21:439–451.

Benbow, M. E., P. S. Barton, M. D. Ulyshen, J. C. Beasley, T. L. DeVault, M. S. Strickland, J. K. Tomberlin, H. R. Jordan, and J. L. Pechal. 2019. Necrobiome framework for bridging decomposition ecology of autotrophically and heterotrophically derived organic matter. Ecological Monographs 89.

Berg, B., and C. McClaugherty. 2014. Plant Litter: Decomposition, Humus Formation, Carbon Sequestration. Springer Berlin Heidelberg, Berlin, Heidelberg.

Bressette, J. W., H. Beck, and V. B. Beauchamp. 2012. Beyond the browse line: complex cascade effects mediated by white-tailed deer. Oikos 121:1749–1760.

Curry, J. P., and O. Schmidt. 2007. The feeding ecology of earthworms – A review. Pedobiologia 50:463–477.

Dávalos, A., E. Simpson, V. Nuzzo, and B. Blossey. 2015. Non-consumptive Effects of Native Deer on Introduced Earthworm Abundance. Ecosystems 18:1029–1042.

De Wandeler, H., R. Sousa-Silva, E. Ampoorter, H. Bruelheide, M. Carnol, S. M. Dawud, G. Dănilă, L. Finer, S. Hättenschwiler, M. Hermy, B. Jaroszewicz, F.-X. Joly, S. Müller, M. Pollastrini, S. Ratcliffe, K. Raulund-Rasmussen, F. Selvi, F. Valladares, K. Van Meerbeek, K. Verheyen, L. Vesterdal, and B. Muys. 2016. Drivers of earthworm incidence and abundance across European forests. Soil Biology and Biochemistry 99:167–178.

Fu, X., F. Yang, J. Wang, Y. Di, X. Dai, X. Zhang, and H. Wang. 2015. Understory vegetation leads to changes in soil acidity and in microbial communities 27years after reforestation. Science of The Total Environment 502:280–286.

Jordan, D., V. C. Hubbard, F. Ponder Jr, and E. C. Berry. 2000. The influence of soil compaction and the removal of organic matter on two native earthworms and soil properties in an oak-hickory forest. Biology and Fertility of Soils 31:323–328.

Kern, C. C., R. A. Montgomery, P. B. Reich, and T. F. Strong. 2013. Canopy gap size influences niche partitioning of the ground-layer plant community in a northern temperate forest. Journal of Plant Ecology 6:101–112.

Kern, C. C., R. A. Montgomery, P. B. Reich, and T. F. Strong. 2014. Harvest-created canopy gaps increase species and functional trait diversity of the forest ground-layer community. Forest Science 60:335–344.

Lefcheck, J. S. 2016. PIECEWISESEM : Piecewise structural equation modelling in R for ecology, evolution, and systematics. Methods in Ecology and Evolution 7:573–579.

Liu, Z., E. Duan, and W. Gao. 2009. Influences of leaf litter replacement on soil biochemical characteristics of main planted forests in Qinling Mountains of China. Frontiers of Agriculture in China 3:346–352.

McAlpine, D. F., M. C. Sollows, G. Sreedharan, G. F. M. Jongsma, and J. W. Reynolds. 2025. An apparent association between North American moose and a native earthworm. Ecology 106:e70146.

Nachtergale, L., K. Ghekiere, A. De Schrijver, B. Muys, S. Luyssaert, and N. Lust. 2002. Earthworm biomass and species diversity in windthrow sites of a temperate lowland forest. Pedobiologia 46:440–451.

Pérez-Izquierdo, L., C. Ménival, P. Baldrian, V. Baldy, C. Biryol, R. Gros, A. Hereş, Y. Le Bagousse-Pinguet, T. Martinovic, I. C. Petritan, S. Sánchez-Moreno, M. Santonja, and J. Curiel Yuste. 2025. Impacts of Canopy Disturbances by Tree Logging on Soil Biota Increase With Organism Size. Global Change Biology 31:e70287.

Ramirez, J. I., P. A. Jansen, J. Den Ouden, L. Moktan, N. Herdoiza, and L. Poorter. 2020. Above- and below-ground cascading effects of wild ungulates in temperate forests. Ecosystems 24:153–167.

Rearick, D., L. Kintz, K. L. Burke, and T. S. Ransom. 2011. Effects of white-tailed deer on the native earthworm, Eisenoides carolinensis, in the southern Appalachian Mountains, USA. Pedobiologia 54:S173–S180.

Reed, S. P., D. R. Bronson, J. A. Forrester, L. M. Prudent, A. M. Yang, A. M. Yantes, P. B. Reich, and L. E. Frelich. 2023. Linked disturbance in the temperate forest: Earthworms, deer, and canopy gaps. Ecology 104:e4040.

Ribeiro, L. G., M. Melo-Dias, B. M. P. Saraiva, N. Villar, and M. Galetti. 2025. Belowground effects of ground-dwelling large herbivores in forest ecosystems. Journal of Animal Ecology:1365-2656.70193.

Sayer, E. J. 2006. Using experimental manipulation to assess the roles of leaf litter in the functioning of forest ecosystems. Biological Reviews 81:1.

Singh, J., M. Schädler, W. Demetrio, G. G. Brown, and N. Eisenhauer. 2019. Climate change effects on earthworms - a review. Soil Organisms:114–138.

Tong, R., B. Ji, G. G. Wang, C. Lou, C. Ma, N. Zhu, W. Yuan, and T. Wu. 2024. Canopy gap impacts on soil organic carbon and nutrient dynamic: a meta-analysis. Annals of Forest Science 81:12.

Wardle, D. A. 1992. A comparative assessment of factors which influence microbial biomass carbon and nitrogen levels in soil. Biological Reviews 67:321–358.

Whalen, J. K., and C. Costa. 2003. Linking spatio-temporal dynamics of earthworm populations to nutrient cycling in temperate agricultural and forest ecosystems. Pedobiologia 47:801–806.

Wu, R., X. Cheng, and H. Han. 2019. The Effect of Forest Thinning on Soil Microbial Community Structure and Function. Forests 10:352.

Xiong, S., and C. Nilsson. 1999. The effects of plant litter on vegetation: a meta-analysis. Journal of Ecology 87:984–994.

Xu, S., L. L. Liu, and E. J. Sayer. 2013. Variability of above-ground litter inputs alters soil physicochemical and biological processes: a meta-analysis of litterfall-manipulation experiments. Biogeosciences 10:7423–7433.

Zhang, D., Y. Zhao, H. Qi, L. Shan, G. Chen, and T. Ning. 2024a. Effects of Micro-Topography and Vegetation on Soil Moisture on Fixed Sand Dunes in Tengger Desert, China. Plants 13:1571.

Zhang, X., P. Heděnec, K. Yue, X. Ni, X. Wei, Z. Chen, J. Yang, and F. Wu. 2024b. Global forest gaps reduce litterfall but increase litter carbon and phosphorus release. Communications Earth & Environment 5:288.

Zhu, J., D. Lu, and W. Zhang. 2014. Effects of gaps on regeneration of woody plants: a meta-analysis. Journal of Forestry Research 25:501–510.
