# Supplementary material for: Native earthworms and roe deer in temperate forests: Friends in the sun, foes in the shade
Source: Ecology. 2026 Sep 22;107(9):e70520. doi: 10.1002/ecy.70520 (PMC13597149; doi:10.1002/ecy.70520)
Supplement: Supplementary file 2 [file ECY-107-e70520-s002.pdf]

Appendix S3

Native earthworms and roe deer in temperate forests: Friends in the sun, foes in the shade

Ludwig Lettenmaier, Nico Eisenhauer, Simone Cesarz, Christian Ammer, Anna Bucharova, Sebastian Dittrich, Ronja Hausmann, Daniel Kraus, Angelika Kölbl, Soumen Mallick, Giorgi Mamadashvili, Atle Mysterud, Kerstin Pierick, Rike Schwarz, Jörg Müller, Christian Ristok

Ecology

Supplementary analyses for soil microbial biomass and litter depth

We analyzed treatment effects on soil microbial biomass and litter depth using linear mixed-effects models implemented in *lme4* (Bates et al. 2015). For each response variable, we fitted models including canopy condition (closed canopy vs. gap), roe deer treatment (absence vs. presence), and their interaction and either total earthworm biomass or total earthworm density as fixed effects. Forest patch was included as a random intercept to account for the paired and hierarchical sampling design. Soil pH and soil water content were included as continuous covariates and were standardized prior to analysis. We evaluated the canopy × roe deer interaction by comparing full and reduced models (with vs. without the interaction term) using likelihood ratio tests and AIC. As the interaction terms were not significant and did not improve model fit, we retained reduced models without interactions for inference.

Table S1: Results of linear mixed-effects models testing the effects of canopy condition (canopy gap vs. closed-canopy), roe deer (presence vs. absence), soil pH, soil water content, and either total earthworm biomass or total earthworm density on soil microbial biomass and litter depth.

**Soil microbial biomass**							
**Fixed effect**	**Estimate**	**SE**	**df**	**t-value**	**p-value**	$_{marg}R^2$	$_{cond}R^2$
						0.40	0.54
(Intercept)	1321.652	42.488	139	31.107	<0.001		
Canopy gap	-289.872	53.984	88	-5.370	<0.001		
Roe deer presence	-48.059	37.474	74	-1.282	0.204		
Total earthworm biomass	-5.599	6.637	142	-0.844	0.400		
scale(Soil pH)	118.431	25.176	120	4.704	<0.001		
scale(Soil water content)	125.917	24.484	144	5.143	<0.001		
**Fixed effect**	**Estimate**	**SE**	**df**	**t-value**	**p-value**	$_{marg}R^2$	$_{cond}R^2$
						0.41	0.55
(Intercept)	1362.855	45.343	140	30.056	<0.001		
Canopy gap	-270.314	54.313	93	-4.977	<0.001		
Roe deer presence	-48.219	36.799	73	-1.310	0.194		
Total earthworm density	-6.706	3.292	142	-2.037	0.043		
scale(Soil pH)	120.037	24.739	119	4.852	<0.001		
scale(Soil water content)	134.274	24.607	143	5.457	<0.001		
**Litter depth**							
**Fixed effect**	**Estimate**	**SE**	**df**	**t-value**	**p-value**	$_{marg}R^2$	$_{cond}R^2$
						0.16	0.41
(Intercept)	3.932	0.270	139	14.571	<0.001		
Canopy gap	-1.108	0.349	91	-3.174	0.002		
Roe deer presence	-0.461	0.230	75	-2.006	0.048		
Total earthworm biomass	-0.086	0.042	140	-2.071	0.040		
scale(Soil pH)	-0.262	0.161	125	-1.628	0.106		
scale(Soil water content)	0.055	0.155	144	0.359	0.720		
**Fixed effect**	**Estimate**	**SE**	**df**	**t-value**	**p-value**	$_{marg}R^2$	$_{cond}R^2$
						0.16	0.41
(Intercept)	3.964	0.292	141	13.577	<0.001		
Canopy gap	-1.032	0.357	97	-2.895	0.005		
Roe deer presence	-0.417	0.228	74	-1.829	0.071		
Total earthworm density	-0.039	0.021	139	-1.879	0.062		
scale(Soil pH)	-0.279	0.161	124	-1.734	0.085		
scale(Soil water content)	0.080	0.158	144	0.509	0.612		

Note: For each response variable, separate models were fitted including either total earthworm biomass or total earthworm density as a continuous predictor, canopy condition (canopy gap vs. closed-canopy), roe deer (presence vs. absence), scaled soil pH, scaled soil water content, and forest patch as a random intercept. Estimates, standard errors (SE), denominator degrees of freedom (df), t-values, p-values, marginal R^2, and conditional R^2 are shown.

Results

Soil microbial biomass was not affected by the total earthworm biomass, whereas it was reduced by higher total earthworm density. In both models (including either total earthworm biomass or density as explanatory variable), canopy gaps strongly reduced soil microbial biomass, while roe deer presence had no significant effect (Table S1). Litter depth was significantly reduced by earthworm biomass, whereas it was only marginally significantly reduced by total earthworm density. Canopy gaps reduced litter depth in both models (including either total earthworm biomass or density). Roe deer presence reduced litter depth in the model that included earthworm biomass, but not in the model that included earthworm density (Table S1).

References

Bates, D., M. Mächler, B. Bolker, and S. Walker. 2015. Fitting linear mixed-effects models using lme4. Journal of Statistical Software 67.
