# Supplementary material for: Native earthworms and roe deer in temperate forests: Friends in the sun, foes in the shade
Source: Ecology. 2026 Sep 22;107(9):e70520. doi: 10.1002/ecy.70520 (PMC13597149; doi:10.1002/ecy.70520)
Supplement: Supplementary file 3 [file ECY-107-e70520-s003.pdf]

Appendix S2

Native earthworms and roe deer in temperate forests: Friends in the sun, foes in the shade

Ludwig Lettenmaier, Nico Eisenhauer, Simone Cesarz, Christian Ammer, Anna Bucharova, Sebastian Dittrich, Ronja Hausmann, Daniel Kraus, Angelika Kölbl, Soumen Mallick, Giorgi Mamadashvili, Atle Mysterud, Kerstin Pierick, Rike Schwarz, Jörg Müller, Christian Ristok

Ecology

Table S1: Summary of earthworm biomass (g) and number of individuals and across treatments.

**Total earthworms**								
	Biomass	Individuals						
Σ(sd)	559.68 (±3.32)	1,509 (±6.92)						
mean	3.73	10.06						
range	0–18.79	0–49						
**Treatment**	**Closed canopy and roe deer presence**		**Closed canopy and roe deer absence**		**Gap and roe deer presence**		**Gap and roe deer absence**	
	Biomass	Individuals	Biomass	Individuals	Biomass	Individuals	Biomass	Individuals
Σ	144.79 (±2.34)	403 (±4.17)	220.75 (±4.08)	533 (±6.51)	102.69 (±4.08)	323 (±10.68)	91.45 (±2.86)	250 (±5.71)
mean(sd)	2.68	7.46	4.09	9.87	3.15	15.38	4.35	11.90
range	0–9.8	0–17	0–18.79	0–34	0.81–11.41	4–49	0.8–11.38	2–28
**Life stage**								
	**Adult**		**Juvenile**					
	Biomass	Individuals	Biomass	Individuals				
Σ(sd)	258.66 (±2.44)	313 (±2)	258.66 (±2.44)	1196.00 (±6.25)				
range	0–10.67	0–12	0–10.67	0–46				
**Treatment**	**Closed canopy and roe deer presence**		**Closed canopy and roe deer absence**		**Gap and roe deer presence**		**Gap and roe deer absence**	
**Adult**								
	Biomass	Individuals	Biomass	Individuals	Biomass	Individuals	Biomass	Individuals
Σ(sd)	59.58 (±1.86)	403 (±4.17)	108.34 (±2.75)	129 (±2.34)	45.91 (±2.61)	50 (±2.38)	44.83 (±2.58)	54 (±1.78)
mean	1.10	7.46	2.01	2.39	2.19	2.22	2.13	2.57
range	0–8.38	0–17	0–10.67	0–12	0–8.68	0–7	0–7.87	0–6
**Juvenile**								
	Biomass	Individuals	Biomass	Individuals	Biomass	Individuals	Biomass	Individuals
Σ(sd)	83.49 (±1.36)	323 (±4.02)	110.72 (±2.11)	404 (±5.33)	55.95 (±1.75)	273 (±10.33)	46.32 (±1.10)	196 (±5.08)
mean	1.55	5.98	2.05	7.48	2.66	13	2.21	9.33
range	0–4.9	0–15	0–11.77	0–29	0.37–5.73	3–46	0.63–4.71	1–22
**Ecological group**								
	**Epigeic**		**Endogeic**		**Anecic**			
	Biomass	Individuals	Biomass	Individuals	Biomass	Individuals		
Σ(sd)	3.30 (±0.12)	8 (±0.28)	130.33 (±0.72)	947 (±4.81)	423.94 (±3.05)	550 (±3.76)		
range	0–0.98	0–2	0–3.7	0–26	0–16.29	0–27		
**Treatment**	**Closed canopy and roe deer presence**		**Closed canopy and roe deer absence**		**Gap and roe deer presence**		**Gap and roe deer absence**	
**Epigeic**								
	Biomass	Individuals	Biomass	Individuals	Biomass	Individuals	Biomass	Individuals
Σ(sd)	0	0	1.44 (±0.03)	3 (±0.23)	0	0	1.86 (±0.26)	5 (±0.62)
mean	0	0	0.12	0.06	0	0	0.09	0.24
range	0–0	0–0	0–0.79	0–1	0–0	0–0	0–0.98	0–2
**Endogeic**								
	Biomass	Individuals	Biomass	Individuals	Biomass	Individuals	Biomass	Individuals
Σ(sd)	35.75 (±0.48)	261 (±3.00)	42.75 (±0.73)	308 (±4.52)	24.19 (±0.87)	214 (±7.12)	27.64 (±0.82)	164 (±4.34)
mean	0.66	4.83	0.79	5.70	1.15	10.19	1.32	7.81
range	0–2.25	0–13	0–3.39	0–18	0.05–3.29	1–26	0.08–3.7	1–15
**Anecic**								
	Biomass	Individuals	Biomass	Individuals	Biomass	Individuals	Biomass	Individuals
Σ(sd)	108.89 (±2.16)	139 (±2.39)	175.48 (±3.84)	222 (±3.66)	77.62 (±2.67)	108 (±5.48)	61.95 (±2.80)	81 (±4.28)
mean	2.02	2.57	3.25	4.11	3.70	5.14	2.95	3.86
range	0–8.55	0–10	0–16.29	0–16	0.03–9.28	1–27	0–10.32	0–20

Notes: Treatments consist of closed canopy and roe deer presence (n = 54), closed canopy and roe deer absence (n = 54), canopy gap and roe deer presence (n = 21), and canopy gap and roe deer absence (n = 21). Values are presented as total sums (Σ), means (± sd), and ranges. Results are shown for total earthworms, separated by life stage (adults and juveniles), and by ecological group (epigeic, endogeic, anecic).
