# Supplementary material for: Native earthworms and roe deer in temperate forests: Friends in the sun, foes in the shade
Source: Ecology. 2026 Sep 22;107(9):e70520. doi: 10.1002/ecy.70520 (PMC13597149; doi:10.1002/ecy.70520)
Supplement: Supplementary file 4 [file ECY-107-e70520-s004.pdf]

Appendix S5

Native earthworms and roe deer in temperate forests: Friends in the sun, foes in the shade

Ludwig Lettenmaier, Nico Eisenhauer, Simone Cesarz, Christian Ammer, Anna Bucharova, Sebastian Dittrich, Ronja Hausmann, Daniel Kraus, Angelika Kölbl, Soumen Mallick, Giorgi Mamadashvili, Atle Mysterud, Kerstin Pierick, Rike Schwarz, Jörg Müller, Christian Ristok

Ecology

Environmental variables

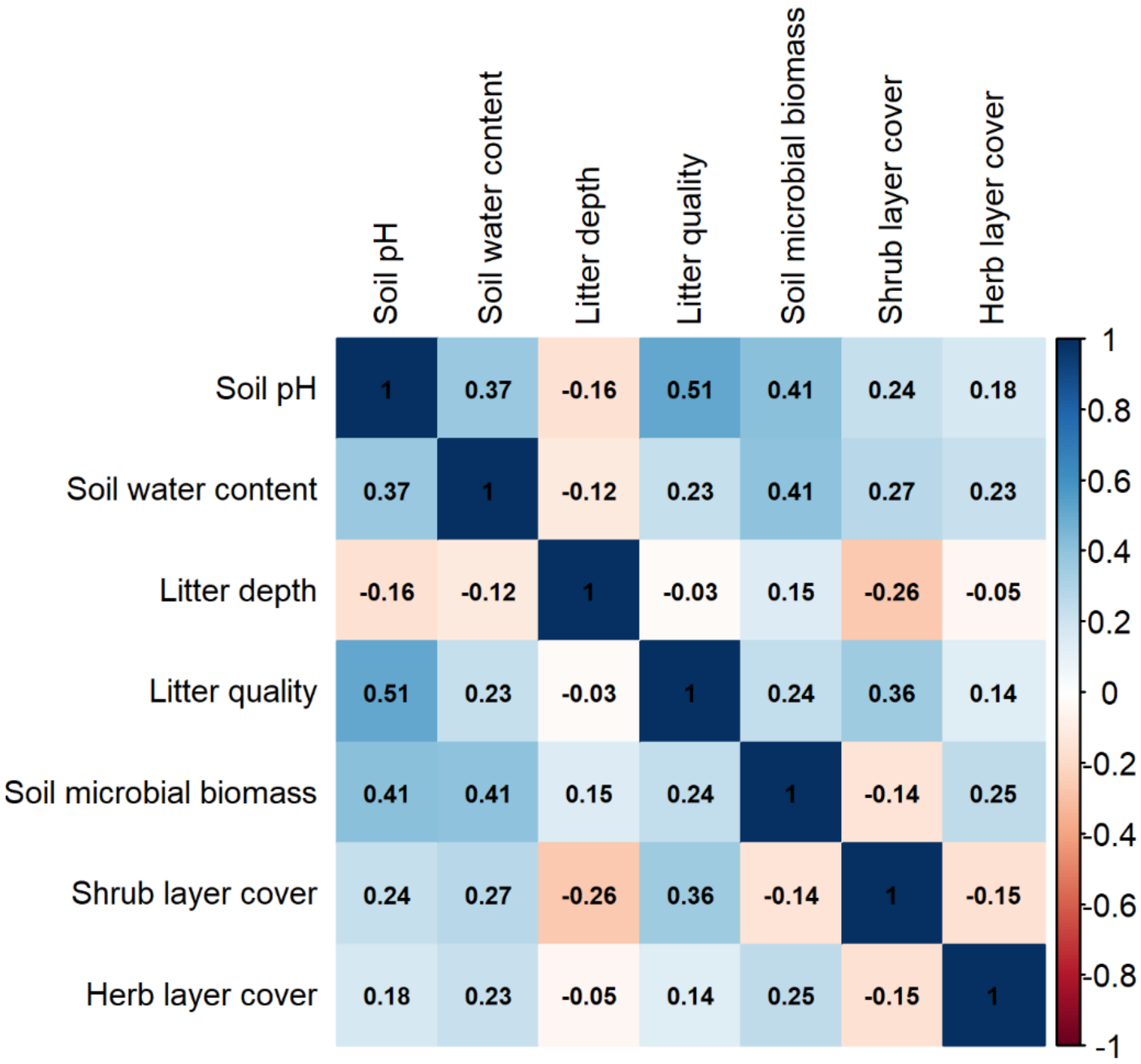

Figure S1: Spearman rank correlation matrix of environmental variables across study plots. Variables include soil pH, soil water content, litter depth, litter quality, soil microbial biomass, shrub layer cover, and herb layer cover. Correlation coefficients are shown with color shading and numeric values; darker colors indicate stronger positive (blue) or negative (red) relationships.
