# Supplementary material for: Native earthworms and roe deer in temperate forests: Friends in the sun, foes in the shade
Source: Ecology. 2026 Sep 22;107(9):e70520. doi: 10.1002/ecy.70520 (PMC13597149; doi:10.1002/ecy.70520)
Supplement: Supplementary file 5 [file ECY-107-e70520-s005.pdf]

Appendix S1

Native earthworms and roe deer in temperate forests: Friends in the sun, foes in the shade

Ludwig Lettenmaier, Nico Eisenhauer, Simone Cesarz, Christian Ammer, Anna Bucharova, Sebastian Dittrich, Ronja Hausmann, Daniel Kraus, Angelika Kölbl, Soumen Mallick, Giorgi Mamadashvili, Atle Mysterud, Kerstin Pierick, Rike Schwarz, Jörg Müller, Christian Ristok

Ecology

Estimation of patch-level litter quality

Trait data on fresh leaf C:N and litter C:N were obtained from the TRY plant trait database (Kattge et al. 2020) for all overstorey tree species recorded in the experimental forest patches (50 m × 50 m). Of the 23 tree species present, fresh leaf C:N values were available for 21, and litter C:N values for 14 species. Fresh and litter C:N values of the same tree species were positively correlated (Pearson's $r = 0.67$, $p = 0.008$). To estimate missing litter C:N values, we fitted a linear model with litter C:N as a function of fresh leaf C:N and predicted values for tree species with unavailable litter data. No predictions were possible for *Sorbus domestica* and *Ulmus glabra* due to the lack of both trait values. These two species, however, contributed only minor proportions to total basal area across patches (mean basal area share: 2.7% for *S. domestica*, 2.4% for *U. glabra*), therefore, their absence is unlikely to bias overall results. For each patch, the basal area proportion of each tree species was calculated. Patch-level litter quality was then estimated as the basal-area-weighted mean litter C:N ratio of the contributing species.

References

Kattge, J., G. Bönisch, S. Díaz, S. Lavorel, I. C. Prentice, P. Leadley, S. Tautenhahn, G. D. A. Werner, T. Aakala, M. Abedi, A. T. R. Acosta, G. C. Adamidis, K. Adamson, M. Aiba, C. H. Albert, J. M. Alcántara, C. Alcázar C, I. Aleixo, H. Ali, B. Amiaud, C. Ammer, M. M. Amoroso, M. Anand, C. Anderson, N. Anten, J. Antos, D. M. G. Apgaua, T. Ashman, D. H. Asmara, G. P. Asner, M. Aspinwall, O. Atkin, I. Aubin, L. Baastrup-Spohr, K. Bahalkeh, M. Bahn, T. Baker, W. J. Baker, J. P. Bakker, D. Baldocchi, J. Baltzer, A. Banerjee, A. Baranger, J. Barlow, D. R. Barneche, Z. Baruch, D. Bastianelli, J. Battles, W. Bauerle, M. Bauters, E. Bazzato, M. Beckmann, H. Beeckman, C. Beierkuhnlein, R. Bekker, G. Belfry, M. Belluau, M. Beloiu, R. Benavides, L. Benomar, M. L. Berdugo-Lattke, E. Berenguer, R. Bergamin, J. Bergmann, M. Bergmann Carlucci, L. Berner, M. Bernhardt-Römermann, C. Bigler, A. D. Bjorkman, C. Blackman, C. Blanco, B. Blonder, D. Blumenthal, K. T. Bocanegra-González, P. Boeckx, S. Bohlman, K. Böhning-Gaese, L. Boisvert-Marsh, W. Bond, B. Bond-Lamberty, A. Boom, C. C. F. Boonman, K. Bordin, E. H. Boughton, V. Boukili, D. M. J. S. Bowman, S. Bravo, M. R. Brendel, M. R. Broadley, K. A. Brown, H. Bruelheide, F. Brumnich, H. H. Bruun, D. Bruy, S. W. Buchanan, S. F. Bucher, N. Buchmann, R. Buitenwerf, D. E. Bunker, J. Bürger, S. Burrascano, D. F. R. P. Burslem, B. J. Butterfield, C. Byun, M. Marques, M. C. Scalon, M. Caccianiga, M. Cadotte, M. Cailleret, J. Camac, J. J. Camarero, C. Campany, G. Campetella, J. A. Campos, L. Cano-Arboleda, R. Canullo, M. Carbognani, F. Carvalho, F. Casanoves, B. Castagneyrol, J. A. Catford, J. Cavender-Bares, B. E. L. Cerabolini, M. Cervellini, E. Chacón-Madrigal, K. Chapin, F. S. Chapin, S. Chelli, S. Chen, A. Chen, P. Cherubini, F. Chianucci, B. Choat, K. Chung, M. Chytrý, D. Ciccarelli, L. Coll, C. G. Collins, L. Conti, D. Coomes, J. H. C. Cornelissen, W. K. Cornwell, P. Corona, M. Coyea, J. Craine, D. Craven, J. P. G. M. Cromsigt, A. Csecserits, K. Cufar, M. Cuntz, A. C. Da Silva, K. M. Dahlin, M. Dainese, I. Dalke, M.

Dalle Fratte, A. T. Dang-Le, J. Danihelka, M. Dannoura, S. Dawson, A. J. De Beer, A. De Frutos, J. R. De Long, B. Dechant, S. Delagrange, N. Delpierre, G. Derroire, A. S. Dias, M. H. Diaz-Toribio, P. G. Dimitrakopoulos, M. Dobrowolski, D. Doktor, P. Dřevojan, N. Dong, J. Dransfield, S. Dressler, L. Duarte, E. Ducouret, S. Dullinger, W. Durka, R. Duursma, O. Dymova, A. E-Vojtkó, R. L. Eckstein, H. Ejtehadi, J. Elser, T. Emilio, K. Engemann, M. B. Erfanian, A. Erfmeier, A. Esquivel-Muelbert, G. Esser, M. Estiarte, T. F. Domingues, W. F. Fagan, J. Fagúndez, D. S. Falster, Y. Fan, J. Fang, E. Farris, F. Fazlioglu, Y. Feng, F. Fernandez-Mendez, C. Ferrara, J. Ferreira, A. Fidelis, B. Finegan, J. Firn, T. J. Flowers, D. F. B. Flynn, V. Fontana, E. Forey, C. Forgiarini, L. François, M. Frangipani, D. Frank, C. Frenette-Dussault, G. T. Freschet, E. L. Fry, N. M. Fyllas, G. G. Mazzochini, S. Gachet, R. Gallagher, G. Ganade, F. Ganga, P. García-Palacios, V. Gargaglione, E. Garnier, J. L. Garrido, A. L. De Gasper, G. Gea-Izquierdo, D. Gibson, A. N. Gillison, A. Giroldo, M. Glasenhardt, S. Gleason, M. Gliesch, E. Goldberg, B. Göldel, E. Gonzalez-Akre, J. L. Gonzalez-Andujar, A. González-Melo, A. González-Robles, B. J. Graae, E. Granda, S. Graves, W. A. Green, T. Gregor, N. Gross, G. R. Guerin, A. Günther, A. G. Gutiérrez, L. Haddock, A. Haines, J. Hall, A. Hambuckers, W. Han, S. P. Harrison, W. Hattingh, J. E. Hawes, T. He, P. He, J. M. Heberling, A. Helm, S. Hempel, J. Hentschel, B. Hérault, A. Hereş, K. Herz, M. Heuertz, T. Hickler, P. Hietz, P. Higuchi, A. L. Hipp, A. Hirons, M. Hock, J. A. Hogan, K. Holl, O. Honnay, D. Hornstein, E. Hou, N. Hough-Snee, K. A. Hovstad, T. Ichie, B. Igić, E. Illa, M. Isaac, M. Ishihara, L. Ivanov, L. Ivanova, C. M. Iversen, J. Izquierdo, R. B. Jackson, B. Jackson, H. Jactel, A. M. Jagodzinski, U. Jandt, S. Jansen, T. Jenkins, A. Jentsch, J. R. P. Jespersen, G. Jiang, J. L. Johansen, D. Johnson, E. J. Jokela, C. A. Joly, G. J. Jordan, G. S. Joseph, D. Junaedi, R. R. Junker, E. Justes, R. Kabzems, J. Kane, Z. Kaplan, T. Kattenborn, L. Kavelenova, E. Kearsley, A. Kempel, T. Kenzo, A. Kerkhoff, M. I. Khalil, N. L. Kinlock, W. D. Kissling, K. Kitajima, T. Kitzberger, R. Kjøller, T. Klein, M. Kleyer, J. Klimešová, J. Klipel, B. Kloeppel, S. Klotz, J. M. H. Knops, T. Kohyama, F. Koike, J. Kollmann, B. Komac, K. Komatsu, C. König, N. J. B. Kraft, K. Kramer, H. Kreft, I. Kühn, D. Kumarathunge, J. Kuppler, H. Kurokawa, Y. Kurosawa, S. Kuyah, J. Laclau, B. Lafleur, E. Lallai, E. Lamb, A. Lamprecht, D. J. Larkin, D. Laughlin, Y. Le Bagousse-Pinguet, G. Le Maire, P. C. Le Roux, E. Le Roux, T. Lee, F. Lens, S. L. Lewis, B. Lhotsky, Y. Li, X. Li, J. W. Lichstein, M. Liebergesell, J. Y. Lim, Y. Lin, J. C. Linares, C. Liu, D. Liu, U. Liu, S. Livingstone, J. Llusià, M. Lohbeck, Á. López-García, G. Lopez-Gonzalez, Z. Lososová, F. Louault, B. A. Lukács, P. Lukeš, Y. Luo, M. Lussu, S. Ma, C. Maciel Rabelo Pereira, M. Mack, V. Maire, A. Mäkelä, H. Mäkinen, A. C. M. Malhado, A. Mallik, P. Manning, S. Manzoni, Z. Marchetti, L. Marchino, V. Marcilio-Silva, E. Marcon, M. Marignani, L. Markesteijn, A. Martin, C. Martínez-Garza, J. Martínez-Vilalta, T. Mašková, K. Mason, N. Mason, T. J. Massad, J. Masse, I. Mayrose, J. McCarthy, M. L. McCormack, K. McCulloh, I. R. McFadden, B. J. McGill, M. Y. McPartland, J. S. Medeiros, B. Medlyn, P. Meerts, Z. Mehrabi, P. Meir, F. P. L. Melo, M. Mencuccini, C. Meredieu, J. Messier, I. Mészáros, J. Metsaranta, S. T. Michaletz, C. Michelaki, S. Migalina, R. Milla, J. E. D. Miller, V. Minden, R. Ming, K. Mokany, A. T. Moles, A. Molnár, J. Molofsky, M. Molz, R. A. Montgomery, A. Monty, L. Moravcová, A. Moreno-Martínez, M. Moretti, A. S. Mori, S. Mori, D. Morris, J. Morrison, L. Mucina, S. Mueller, C. D. Muir, S. C. Müller, F. Munoz, I. H. Myers-Smith, R. W. Myster, M. Nagano, S. Naidu, A. Narayanan, B. Natesan, L. Negoita, A. S. Nelson, E. L. Neuschulz, J. Ni, G. Niedrist, J. Nieto, Ü. Niinemets, R. Nolan, H. Nottebrock, Y. Nouvellon, A. Novakovskiy, The Nutrient Network, K. O. Nystuen, A. O'Grady, K. O'Hara, A. O'Reilly-Nugent, S. Oakley, W. Oberhuber, T. Ohtsuka, R. Oliveira, K. Öllerer, M. E. Olson, V. Onipchenko, Y. Onoda, R. E. Onstein, J. C. Ordonez, N. Osada, I. Ostonen, G. Ottaviani, S. Otto, G. E. Overbeck, W. A. Ozinga, A.

T. Pahl, C. E. T. Paine, R. J. Pakeman, A. C. Papageorgiou, E. Parfionova, M. Pärtel, M. Patacca, S. Paula, J. Paule, H. Pauli, J. G. Pausas, B. Peco, J. Penuelas, A. Perea, P. L. Peri, A. C. Petisco-Souza, A. Petraglia, A. M. Petritan, O. L. Phillips, S. Pierce, V. D. Pillar, J. Pisek, A. Pomogaybin, H. Poorter, A. Portsmuth, P. Poschlod, C. Potvin, D. Pounds, A. S. Powell, S. A. Power, A. Prinzing, G. Puglielli, P. Pyšek, V. Raevel, A. Rammig, J. Ransijn, C. A. Ray, P. B. Reich, M. Reichstein, D. E. B. Reid, M. Réjou-Méchain, V. R. De Dios, S. Ribeiro, S. Richardson, K. Riibak, M. C. Rillig, F. Riviera, E. M. R. Robert, S. Roberts, B. Robroek, A. Roddy, A. V. Rodrigues, A. Rogers, E. Rollinson, V. Rolo, C. Römermann, D. Ronzhina, C. Roscher, J. A. Rosell, M. F. Rosenfield, C. Rossi, D. B. Roy, S. Royer-Tardif, N. Rüger, R. Ruiz-Peinado, S. B. Rumpf, G. M. Rusch, M. Ryo, L. Sack, A. Saldaña, B. Salgado-Negret, R. Salguero-Gomez, I. Santa-Regina, A. C. Santacruz-García, J. Santos, J. Sardans, B. Schamp, M. Scherer-Lorenzen, M. Schleuning, B. Schmid, M. Schmidt, S. Schmitt, J. V. Schneider, S. D. Schowanek, J. Schrader, F. Schrodt, B. Schuldt, F. Schurr, G. Selaya Garvizu, M. Semchenko, C. Seymour, J. C. Sfair, J. M. Sharpe, C. S. Sheppard, S. Sheremetiev, S. Shiodera, B. Shipley, T. A. Shovon, A. Siebenkäs, C. Sierra, V. Silva, M. Silva, T. Sitzia, H. Sjöman, M. Slot, N. G. Smith, D. Sodhi, P. Soltis, D. Soltis, B. Somers, G. Sonnier, M. V. Sørensen, E. E. Sosinski, N. A. Soudzilovskaia, A. F. Souza, M. Spasojevic, M. G. Sperandii, A. B. Stan, J. Stegen, K. Steinbauer, J. G. Stephan, F. Sterck, D. B. Stojanovic, T. Strydom, M. L. Suarez, J. Svenning, I. Svitková, M. Svitok, M. Svoboda, E. Swaine, N. Swenson, M. Tabarelli, K. Takagi, U. Tappeiner, R. Tarifa, S. Tauugourdeau, C. Tavsanoglu, M. Te Beest, L. Tedersoo, N. Thiffault, D. Thom, E. Thomas, K. Thompson, P. E. Thornton, W. Thuiller, L. Tichý, D. Tissue, M. G. Tjoelker, D. Y. P. Tng, J. Tobias, P. Török, T. Tarin, J. M. Torres-Ruiz, B. Tóthmérész, M. Treurnicht, V. Trivellone, F. Trolliet, V. Trotsiuk, J. L. Tsakalos, I. Tsiripidis, N. Tysklind, T. Umehara, V. Usoltsev, M. Vadeboncoeur, J. Vaezi, F. Valladares, J. Vamosi, P. M. Van Bodegom, M. Van Breugel, E. Van Cleemput, M. Van De Weg, S. Van Der Merwe, F. Van Der Plas, M. T. Van Der Sande, M. Van Kleunen, K. Van Meerbeek, M. Vanderwel, K. A. Vanselow, A. Vårhammar, L. Varone, M. Y. Vasquez Valderrama, K. Vassilev, M. Vellend, E. J. Veneklaas, H. Verbeeck, K. Verheyen, A. Vibrans, I. Vieira, J. Villacís, C. Violle, P. Vivek, K. Wagner, M. Waldram, A. Waldron, A. P. Walker, M. Waller, G. Walther, H. Wang, F. Wang, W. Wang, H. Watkins, J. Watkins, U. Weber, J. T. Weedon, L. Wei, P. Weigelt, E. Weiher, A. W. Wells, C. Wellstein, E. Wenk, M. Westoby, A. Westwood, P. J. White, M. Whitten, M. Williams, D. E. Winkler, K. Winter, C. Womack, I. J. Wright, S. J. Wright, J. Wright, B. X. Pinho, F. Ximenes, T. Yamada, K. Yamaji, R. Yanai, N. Yankov, B. Yguel, K. J. Zanini, A. E. Zanne, D. Zelený, Y. Zhao, J. Zheng, J. Zheng, K. Ziemińska, C. R. Zirbel, G. Zizka, I. C. Zo-Bi, G. Zotz, and C. Wirth. 2020. TRY plant trait database – enhanced coverage and open access. Global Change Biology 26:119–188.
