# Supplementary material for: Native earthworms and roe deer in temperate forests: Friends in the sun, foes in the shade
Source: Ecology. 2026 Sep 22;107(9):e70520. doi: 10.1002/ecy.70520 (PMC13597149; doi:10.1002/ecy.70520)
Supplement: Supplementary file 6 [file ECY-107-e70520-s006.pdf]

Appendix S6

Native earthworms and roe deer in temperate forests: Friends in the sun, foes in the shade

Ludwig Lettenmaier, Nico Eisenhauer, Simone Cesarz, Christian Ammer, Anna Bucharova, Sebastian Dittrich, Ronja Hausmann, Daniel Kraus, Angelika Kölbl, Soumen Mallick, Giorgi Mamadashvili, Atle Mysterud, Kerstin Pierick, Rike Schwarz, Jörg Müller, Christian Ristok

Ecology

Table S1: Results of mixed-effects models testing the effects of canopy condition, roe deer, and soil variables on earthworm biomass and density across total communities, life stages, and ecological groups.

		Fixed effect	Estimate	SE	df	t-value	p-value
Total earthworms	Biomass	(Intercept)	1.440	0.063	135	23.002	<0.001
		Canopy gap	0.068	0.119	141	0.574	0.567
		Roe deer presence	-0.182	0.076	75	-2.402	0.019
		scale(Soil pH)	0.089	0.045	119	1.984	0.050
		scale(Soil water content)	0.020	0.044	144	0.444	0.658
		Canopy gap × Roe deer presence	0.255	0.147	80	1.732	0.087
	Density	**Fixed effects**	**Estimate**	**SE**		**z-value**	**p-value**
		(Intercept)	2.234	0.085		26.258	<0.001
		Canopy gap	0.123	0.155		0.795	0.427
		Roe deer presence	-0.253	0.106		-2.389	0.017
		scale(Soil pH)	0.118	0.062		1.917	0.055
		scale(Soil water content)	0.091	0.059		1.546	0.122
		Canopy gap × Roe deer presence	0.446	0.195		2.291	0.022
Life stage	Biomass	**Fixed effect**	**Estimate**	**SE**	**df**	**t-value**	**p-value**
		(Intercept)	0.947	0.071	274	13.304	<0.001
		Lifestage juvenile	0.182	0.093	214	1.950	0.052
		Lifestage adult × Canopy gap	0.056	0.135	281	0.412	0.680
		Lifestage juvenile × Canopy gap	0.105	0.135	281	0.772	0.440
		Lifestage adult × Roe deer presence	-0.235	0.094	221	-2.495	0.013
		Lifestage juvenile × Roe deer presence	-0.119	0.094	221	-1.267	0.206
		Lifestage adult × Canopy gap × Roe deer presence	0.218	0.182	228	1.200	0.231
		Lifestage juvenile × Canopy gap × Roe deer presence	0.183	0.182	228	1.003	0.317
		Lifestage adult × scale(Soil pH)	0.031	0.050	238	0.629	0.530
		Lifestage juvenile × scale(Soil pH)	0.073	0.050	238	1.473	0.142
		Lifestage adult × scale(Soil water content)	0.034	0.050	282	0.677	0.499
		Lifestage juvenile × scale(Soil water content)	0.000	0.050	282	0.009	0.993
	Density	**Fixed effect**	**Estimate**	**SE**		**z-value**	**p-value**
		(Intercept)	0.818	0.120		6.797	<0.001
		Lifestage juvenile	1.140	0.141		8.114	<0.001
		Lifestage adult × Canopy gap	0.059	0.223		0.266	0.790
		Lifestage juvenile × Canopy gap	0.145	0.175		0.828	0.407
		Lifestage adult × Roe deer presence	-0.452	0.174		-2.597	0.009
		Lifestage juvenile × Roe deer presence	-0.200	0.124		-1.611	0.107
		Lifestage adult × Canopy gap × Roe deer presence	0.372	0.315		1.182	0.237

		Lifestage juvenile × Canopy gap × Roe deer presence	0.463	0.228	2.033	0.042
		Lifestage adult × scale(Soil pH)	0.065	0.091	0.710	0.478
		Lifestage juvenile × scale(Soil pH)	0.130	0.068	1.893	0.058
		Lifestage adult × scale(Soil water content)	0.023	0.087	0.258	0.797
		Lifestage juvenile × scale(Soil water content)	0.114	0.066	1.736	0.083
**Ecological group**	**Biomass**	**Fixed effect**	**Estimate**	**SE**	**z-value**	**p-value**
		(Intercept)	1.038	0.126	8.268	<0.001
		Endogeic	-1.307	0.182	-7.189	<0.001
		Anecic × Canopy gap	-0.101	0.225	-0.449	0.653
		Endogeic × Canopy gap	0.484	0.275	1.758	0.079
		Anecic × Roe deer presence	-0.439	0.160	-2.751	0.006
		Endogeic × Roe deer presence	-0.158	0.214	-0.741	0.459
		Anecic × Canopy gap × Roe deer presence	0.629	0.290	2.169	0.030
		Endogeic × Canopy gap × Roe deer presence	-0.045	0.379	-0.12	0.904
		Anecic × scale(Soil pH)	0.215	0.093	2.316	0.021
		Endogeic × scale(Soil pH)	0.01	0.111	0.086	0.931
		Anecic × scale(Soil water content)	0.064	0.087	0.731	0.465
		Endogeic × scale(Soil water content)	0.127	0.107	1.187	0.235
	**Density**	**Fixed effect**	**Estimate**	**SE**	**z-value**	**p-value**
		(Intercept)	1.351	0.114	11.901	<0.001
		Endogeic	0.318	0.140	2.274	0.023
		Anecic × Canopy gap	-0.158	0.215	-0.733	0.463
		Endogeic × Canopy gap	0.273	0.196	1.393	0.164
		Anecic × Roe deer presence	-0.474	0.154	-3.082	0.002
		Endogeic × Roe deer presence	-0.105	0.139	-0.754	0.451
		Anecic × Canopy gap × Roe deer presence	0.742	0.284	2.612	0.009
		Endogeic × Canopy gap × Roe deer presence	0.273	0.253	1.076	0.282
		Anecic × scale(Soil pH)	0.150	0.085	1.765	0.078
		Endogeic × scale(Soil pH)	0.092	0.077	1.198	0.231
		Anecic × scale(Soil water content)	0.052	0.082	0.634	0.526
		Endogeic × scale(Soil water content)	0.130	0.073	1.776	0.076

Notes: Biomass was analyzed using linear mixed-effects models, except for ecological-group analyses, which were fitted using Tweedie generalized linear mixed-effects models. Density was analyzed using negative binomial generalized linear mixed-effects models. Biomass estimates are on the cube-root-transformed scale for LMMs and on the log scale for Tweedie GLMMs. Density estimates are on the log scale. Models included canopy condition, roe deer, scaled soil pH, scaled soil water content, and forest patch as a random effect. In life-stage and ecological-group models, the focal factor was interacted with canopy, roe deer presence, their interaction, and soil covariates. Denominator degrees of freedom are reported for LMMs with t-tests. GLMMs were evaluated using asymptotic z-tests, for which denominator degrees of freedom are not reported. Estimates, standard errors (SE), test statistics, and p-values are shown.

Table S2: Type III Wald χ^2 tests evaluating interaction effects of life stage and ecological group with canopy condition and roe deer on earthworm biomass and density.

		χ^2	df	p-value	$_{marg}R^2$	$_{cond}R^2$
**Life stage**	**Biomass**				0.11	0.24
	(Intercept)	178.000	1	<0.001		
	Life stage	3.802	1	0.051		
	Life stage × Canopy	0.695	2	0.707		
	Life stage × Roe deer	7.782	2	0.020		
	Life stage × scale(Soil pH)	2.292	2	0.318		
	Life stage × scale(Soil water content)	0.468	2	0.792		
	Life stage × Canopy × Roe deer	2.406	2	0.300		
	**Density**				0.49	0.57
	(Intercept)	46.205	1	<0.001		
	Life stage	65.829	1	<0.001		
	Life stage × Canopy	0.709	2	0.702		
	Life stage × Roe deer	9.276	2	0.010		
	Life stage × scale(Soil pH)	3.732	2	0.155		
	Life stage × scale(Soil water content)	3.014	2	0.222		
	Life stage × Canopy × Roe deer	5.445	2	0.066		
**Ecological group**	**Biomass**				0.36	0.51
	(Intercept)	68.352	1	<0.001		
	Ecological group	51.686	1	<0.001		
	Ecological group × Canopy	3.684	2	0.159		
	Ecological group × Roe deer	8.065	2	0.018		
	Ecological group × scale(Soil pH)	5.551	2	0.062		
	Ecological group × scale(Soil water content)	1.672	2	0.433		
	Ecological group × Canopy × Roe deer	4.733	2	0.094		
	**Density**				0.22	0.36
	(Intercept)	141.634	1	<0.001		
	Ecological group	5.171	1	0.023		
	Ecological group × Canopy	2.901	2	0.234		
	Ecological group × Roe deer	10.013	2	0.007		
	Ecological group × scale(Soil pH)	3.823	2	0.148		
	Ecological group × scale(Soil water content)	3.250	2	0.197		
	Ecological group × Canopy × Roe deer	7.854	2	0.020		

Note: Linear and generalized linear mixed-effects models included interactions between life stage or ecological group and canopy condition, roe deer, and their interaction. Soil pH and soil water content were included as scaled covariates (interacted with the focal factor), and forest patch was included as a random effect. χ^2 statistics, degrees of freedom (df), p-values are shown and marginal and conditional R^2 values are shown.

Table S3: Planned contrasts among four treatment combinations testing context-dependent effects of canopy condition and roe deer presence on earthworm biomass and density across life stages and ecological groups.

Contrast	Estimate	SE	95% CI lower	95% CI upper	z-value	p-value
**Adult biomass**						
Closed canopy: roe deer presence - absence	-0.235	0.094	-0.419	-0.050	-2.495	0.050
Canopy gap: roe deer presence - absence	-0.016	0.153	-0.316	0.283	-0.107	1.000
Roe deer absence: canopy gap - closed canopy	0.056	0.135	-0.210	0.321	0.412	1.000
Roe deer presence: canopy gap - closed canopy	0.274	0.142	-0.004	0.552	1.933	0.160
**Juvenile biomass**						
Closed canopy: roe deer presence - absence	-0.119	0.094	-0.304	0.065	-1.267	0.615
Canopy gap: roe deer presence - absence	0.063	0.153	-0.236	0.363	0.414	0.880
Roe deer absence: canopy gap - closed canopy	0.105	0.135	-0.161	0.370	0.772	0.880
Roe deer presence: canopy gap - closed canopy	-0.287	0.142	0.009	0.565	-2.024	0.172
**Adult density**						
Closed canopy: roe deer presence - absence	-0.452	0.174	-0.793	-0.111	-2.597	0.038
Canopy gap: roe deer presence - absence	-0.080	0.257	-0.584	0.423	-0.313	1.000
Roe deer absence: canopy gap - closed canopy	0.059	0.223	-0.378	0.496	0.266	1.000
Roe deer presence: canopy gap - closed canopy	0.431	0.249	-0.057	0.919	1.730	0.251
**Juvenile density**						
Closed canopy: roe deer presence - absence	-0.200	0.124	-0.443	0.043	-1.611	0.321
Canopy gap: roe deer presence - absence	0.263	0.187	-0.103	0.630	1.409	0.321
Roe deer absence: canopy gap - closed canopy	0.145	0.175	-0.198	0.489	0.828	0.407
Roe deer presence: canopy gap - closed canopy	0.609	0.183	0.249	0.968	3.319	0.004
**Anecic biomass**						
Closed canopy: roe deer presence - absence	-0.439	0.160	-0.752	-0.126	-2.751	0.024
Canopy gap: roe deer presence - absence	0.190	0.238	-0.276	0.657	0.799	0.849
Roe deer absence: canopy gap - closed canopy	-0.101	0.225	-0.543	0.341	-0.449	0.849
Roe deer presence: canopy gap - closed canopy	0.528	0.240	0.058	0.998	2.201	0.083
**Endogeic biomass**						
Closed canopy: roe deer presence - absence	-0.158	0.214	-0.577	0.260	-0.741	0.917
Canopy gap: roe deer presence - absence	-0.204	0.305	-0.802	0.395	-0.667	0.917
Roe deer absence: canopy gap - closed canopy	0.484	0.275	-0.056	1.023	1.758	0.315
Roe deer presence: canopy gap - closed canopy	0.438	0.303	-0.156	1.032	1.446	0.444
**Anecic density**						
Closed canopy: roe deer presence - absence	-0.474	0.154	-0.776	-0.173	-3.082	0.008
Canopy gap: roe deer presence - absence	0.267	0.235	-0.193	0.728	1.138	0.510
Roe deer absence: canopy gap - closed canopy	-0.158	0.215	-0.580	0.264	-0.733	0.510
Roe deer presence: canopy gap - closed canopy	0.584	0.225	0.142	1.025	2.591	0.029
**Endogeic density**						
Closed canopy: roe deer presence - absence	-0.105	0.139	-0.378	0.167	-0.754	0.832
Canopy gap: roe deer presence - absence	0.168	0.206	-0.237	0.572	0.813	0.832
Roe deer absence: canopy gap - closed canopy	0.273	0.196	-0.111	0.656	1.393	0.491
Roe deer presence: canopy gap - closed canopy	0.545	0.205	0.143	0.947	2.657	0.032

Notes: Planned contrasts were derived from (generalized) linear effect models. Estimates represent differences between treatment levels within canopy or roe deer context. Standard errors (SE), 95% confidence intervals, z-values, and Holm-adjusted p-values are reported.

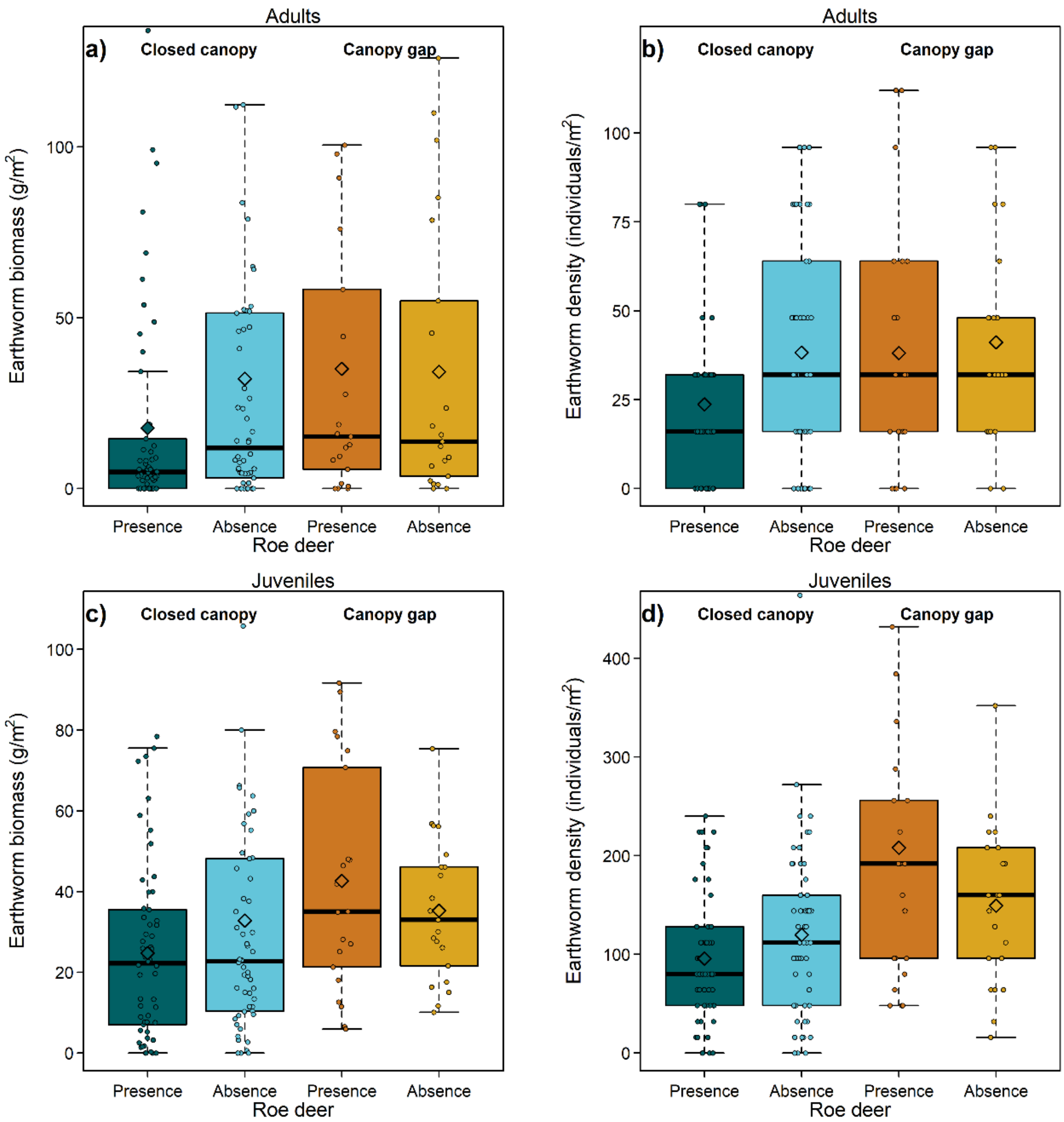

Figure S1: Adult earthworm **(a)** biomass (g/m²) and **(b)** density (individuals/m²), and juvenile earthworm **(c)** biomass (g/m²) and **(d)** density (individuals/m²), across canopy condition (closed canopy vs. canopy gap) and roe deer (absence vs. presence) treatments. Roe deer presence and absence plots were established in canopy gaps (n = 21 per treatment) and closed canopy forest patches (n = 54 per treatment). Boxplots show medians, interquartile ranges, and whiskers. Circles show individual plots, and diamonds indicate group means. Statistical tests for planned contrasts among treatment combinations are reported in Table S3.

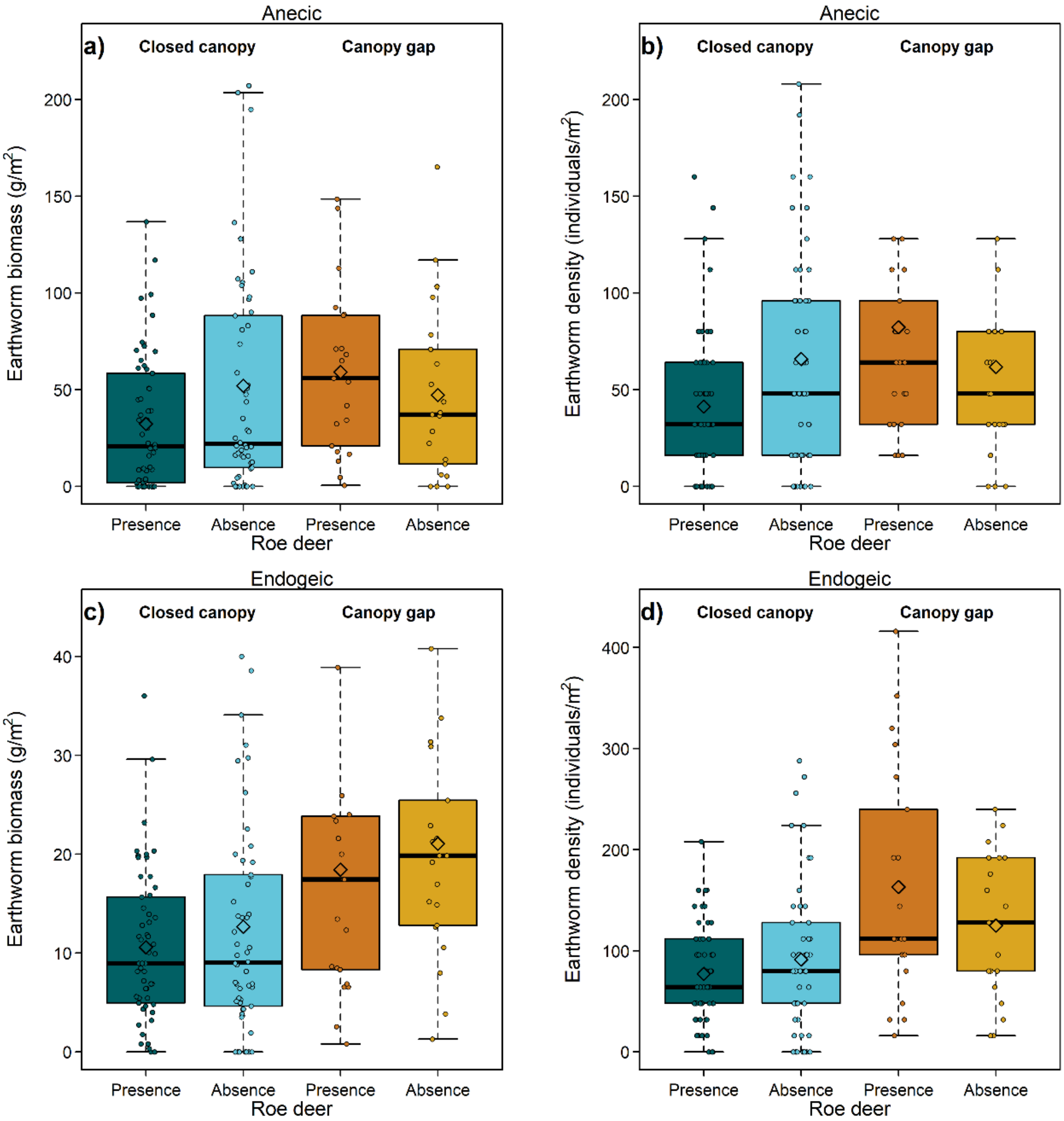

Figure S2: Anecic earthworm **(a)** biomass (g/m²) and **(b)** density (individuals/m²), and endogeic earthworm **(c)** biomass (g/m²) and **(d)** density (individuals/m²), across canopy condition (closed canopy vs. gap) and roe deer (absence vs. presence) treatments. Roe deer presence and absence plots were established in canopy gaps (n = 21 per treatment) and closed canopy forest patches (n = 54 per treatment). Boxplots show medians, interquartile ranges, and whiskers. Circles show individual plots, and diamonds indicate group means. Statistical tests for planned contrasts among treatment combinations are reported in Table S3.
